# Reduced circulating CD63^+^ extracellular vesicle levels associate with atherosclerosis in hypercholesterolaemic mice and humans

**DOI:** 10.1186/s12933-024-02459-w

**Published:** 2024-10-17

**Authors:** Brachyahu M. Kestecher, Krisztina Németh, Sayam Ghosal, Nabil V. Sayour, Tamás G. Gergely, Bernadett R. Bodnár, András I. Försönits, Barbara W. Sódar, Johannes Oesterreicher, Wolfgang Holnthoner, Zoltán V. Varga, Zoltán Giricz, Péter Ferdinandy, Edit I. Buzás, Xabier Osteikoetxea

**Affiliations:** 1 Institute of Genetics, Cell- and Immunobiology, Semmelweis University, Budapest, Hungary; 2 HUN-REN-SU Translational Extracellular Vesicle Research Group, Budapest, Hungary; 3 HCEMM-SU Extracellular Vesicle Research Group, Budapest, Hungary; 4 Department of Pharmacology and Pharmacotherapy, Semmelweis University, Budapest, Hungary; 5 Pharmahungary Group, Szeged, Hungary; 6 Ludwig-Boltzmann-Institute for Traumatology, The Research Centre in Cooperation with AUVA, Vienna, Austria; 7 Austrian Cluster for Tissue Regeneration, Vienna, Austria

## Abstract

**Aims:**

The association and co-isolation of low-density lipoproteins (LDL) and extracellular vesicles (EVs) have been shown in blood plasma. Here we explore this relationship to better understand the role of EVs in atherogenesis.

**Methods and results:**

Wild type (WT), PCSK9^−/−^, and LDLR^−/−^ C57BL/6 mice were used in this study. Eleven week-old male mice were fed high-fat diet (HFD) for 12 weeks or kept on normal diet until old age (22-months). Cardiac function was assessed by ultrasound, cholesterol was quantified with a colorimetric kit and circulating EVs were measured using flow cytometry. Plaques were analysed post-mortem using Oil-Red-O staining of the aortic arch. EVs were measured from platelet free blood plasma samples of normal and hypercholesterolaemic clinical patients. Based on annexin V and CD63 staining, we found a significant increase in EV levels in LDLR^−/−^ and PCSK9^−/−^ mice after HFD, but CD81 showed no significant change in either group. There was no significant change in plaque formation after HFD. PCSK9^−/−^ mice show a favourable cardiac function after HFD. Blood cholesterol levels progressively increased during HFD, with LDLR^−/−^ mice showing high levels while PCSK9^−/−^ were significantly lowered compared to WT animals. In mice at old age, similar cholesterol levels were observed as in young mice. In old age, LDLR^−/−^ mice showed significantly increased plaques. At old age, ejection fraction was decreased in all groups of mice, as were CD63^+^ EVs. Similarly to mice, in patients with hypercholesterolaemia, CD63^+^ EVs were significantly depleted.

**Conclusions:**

This research demonstrates an inverse relationship between circulating EVs and cholesterol, making EVs a potential marker for cardiovascular disease (CVD). HFD causes reduced cardiac function, but atherosclerotic development is slowly progressing in hypercholesterolaemic models and only observed with old animals. These results also bring further evidence for the benefit of using of PCSK9 inhibitors as therapeutic agents in CVD.

**Supplementary Information:**

The online version contains supplementary material available at 10.1186/s12933-024-02459-w.

## Methods

### Mice

All mouse experiments followed procedures outlined by the Council Directive of the European Union (86/609/EEC), and prior to experimental work, approval was granted by Semmelweis University’s Institutional Animal Care and Use Committee (PE/EA/1363-8/2019). Ten week old male, wild type (WT), PCSK9^−/−^ and LDLR^−/−^ mice were used with groups (*n* = 6, all were C57BL/6J background). Mice were supplied by the Jackson Laboratory (JAX catalogue numbers: 000664, 005993 and 002207, Bar Harbor, USA). Mice were maintained at the animal facility of the Institute of Genetics, Cell- and Immunobiology, with a normal light cycle (12 h light– 12 h dark) and were given free access to food and water. Blood was taken from the retrobulbar venous plexus at experimental start point (week 10) while animals continued normal feeding. At week 11, mice were starved for 5 h after which retrobulbar venous plexus blood was collected and mice were fed high fat diet (HFD) consisting of 45 kcal% fat (catalogue # D12451, Research Diets, New Brunswick, USA) from this point forward for 12 weeks.

At the midpoint of HFD (week + 6), mice were starved for 5 h after which retrobulbar venous plexus blood was collected. At 23 weeks, mice were anesthetised using isoflurane (1 mL/100 g body mass) and sacrificed through terminal bleeding via the inferior vena cava. Mice were weighed on a weekly basis from age 10 weeks through week 23.

For long-term aging experiments, male, WT, PCSK9^−/−^ and LDLR^−/−^ mice were used with group sizes *n* = 6 (all were C57BL/6J background). All mice were kept on normal chow diet for 22-months. At 22-months, surviving mice were anesthetised using isoflurane (1 mL/100 g body mass) and sacrificed through terminal bleeding via the inferior vena cava. Mice were weighed at termination.

### Genotyping of mice

Genotyping confirmation was performed every 12–18 months or when a new breeding cage was set up, to ensure homozygous breeding cages and offspring. Typically, 2 females and 1 male from each knock-out (KO) strain was genotyped simultaneously, plus 1 WT animal as a negative control. Fifty bp DNA ladder (catalogue # SM0373, Thermo Scientific, Waltham, USA) was used. PCR primers and PCR protocols of the Jackson Laboratory were followed. For PCSK9^−/−^ genotyping protocol 27,858: Standard PCR Assay - Pcsk9 < tm1Jdh > Version 4.2 was followed and for LDLR^−/−^ genotyping protocol 27,075: Standard PCR Assay - Ldlr < tm1Her>-alternate 1 Version 1.2 was followed.

### Human samples

All human platelet free plasma (PFP) samples were prepared from venous blood samples taken from patients admitted to the Semmelweis Városmajor Clinic. The patients were emergency patients with chest pain diagnosed not to have an acute cardiac ischemic event. Approval was granted by Semmelweis University’s Regional and Institutional Committee of Science and Research Ethics (**67/2022**). Blood samples from male patients were used for measurement, with patient data displayed in Supplementary Table 1. Group A were patients with normal cholesterol levels and Group B were patients with hypercholesterolaemia (*n* = 6). TC, LDL-C, high-density lipoprotein cholesterol (HDL-C), triglycerides, Glucose, and HbA1C were all measured using the DxC 700 AU (Beckmann Coulter, Brea, USA).

### Mouse blood collection, handling, and storage

Blood was collected from the retrobulbar venous plexus using Corning^®^ Pasteur pipettes L 5 3/4 in. (146 mm), standard tip (catalogue # CLS7095B5X-1000EA, Merck Sigma, Darmstadt, Germany) on all occasions except when mice were sacrificed. When sacrificing the mice, blood was taken from the inferior vena cava using a syringe and needle for careful extraction. All blood samples were gently mixed with 15% ACD-A anti-coagulant and processed within two hours from collection. Blood was centrifuged 2,500 g for 15 min at 4 °C, platelet poor plasma (PPP) was removed, 45 µL was taken and left at 4 °C for cholesterol measurements. For EV flow cytometry measurements, the rest of the PPP was re-spun 2,500 g for 15 min at 4 °C– to produce PFP, which was aliquoted into 1.5 mL Eppendorf tubes, diluted 1:1 in 0.1 μm filtered 0.9% NaCl (saline) solution, flash frozen in liquid nitrogen and stored at − 80 °C.

### Assessment of cardiovascular function

All cardiovascular parameters were measured before termination of animals on the final day of experimental timeline, as described earlier [1]. A Vevo 3100 high-resolution in vivo imaging system (Fujifilm VisualSonics, Toronto, Canada) was used with an ultrahigh frequency transducer (MX400, 30 MHz, 55 fps) and evaluation was performed using VevoLAB software (version 5.6.1, Fujifilm VisualSonics, Toronto, Canada). Animals were anesthetized with isoflurane (for induction: 5 V/V% in O_2_; for maintenance: 2 V/V% in O_2_), with spontaneous breathing. Throughout the echocardiographic measurements, body temperature was kept at 37 ± 0.5°C, and electrocardiographic activity was obtained continuously. Chest hair was removed, and two-dimensional cines were obtained from the long-, short-, and apical four-chamber views of the heart (LAX, SAX, and APIC4, respectively). Left ventricular end-systolic and end-diastolic volumes (LVESV and LVEDV, respectively, derived from the rotational volumes of the left ventricular trace at diastole and systole, around the long axis line of the spline), left ventricular stroke volume (LVSV), left ventricular ejection fraction (LVEF) and cardiac output (CO) measurements were obtained from the LAX view. Left ventricular diameters in end-systole and end-diastole (LVESD, LVEDD, respectively), left ventricular fractional shortening (FS), left ventricular mass (LV mass), and left ventricular anterior or posterior wall thicknesses in systole and diastole (LVAWTs, LVAWTd, LVPWTs, and LVPWTd, respectively) were acquired from the SAX view. Relative wall thickness (RWT) was calculated as 2*LVPWTd/LVEDD; left ventricular remodelling index (LVRi) was calculated as LV mass/LVEDD. Iso-volumetric relaxation time (IVRT), and E/e’ ratio (where E: peak transmitral blood flow velocity in early diastole measured by pulsed wave Doppler imaging; and where e’: mitral annular velocity in early diastole assessed by tissue Doppler imaging) were derived from the APIC4 view, indicative for diastolic function.

### Quantification of aortic atherosclerotic plaques

The aortic arches were isolated after termination of the mice using a stereomicroscope. Plaques were stained and imaged following an earlier protocol [2]. Here, aortic arches were stained using Oil-Red-O (Catalogue # 31170, Serva, Heidelberg, Germany, ) with a 70% ethanol wash prior to staining and an 80% ethanol wash post stain. Stained specimens were stored in PBS until imaged. Images were taken using the S9D microscope with objective lens 10450528 using the attached FLEXACAM C1 camera (Leica, Wetzlar, Germany).

ImageJ software was used to analyse the Oil-Red-O^+^ area/whole tissue ratio (%) of the plaques. Wand tool was used to select and measure the total isolated whole tissue area. The following steps were used to determine the plaques present on each isolated aortic arch:

1. The “Image” tab was selected and from there “colour” and then “split channels” was used.
2. The “process” tab was used to get the “image calculator” where red was subtracted from green.
3. In the “image” tab, “adjust” and then “threshold” was selected (for Oil-Red-O^+^ particles) (each determined by eyes of two independent researchers).
4. Here, watershed of the image (of Oil-Red-O^+^ particles) was performed by selecting the process” tab and then “binary” and finally watershed.
5. After which, the total particle area was obtained using the “analyse” tab and selecting “measure particles”, ensuring any size or shaped particle was measured (0-infinity, 0.0–1.0).

All images and groups were initially randomised and blinded by a first researcher before independent analysis by two separate researchers.

### Quantification of mouse blood cholesterol levels

Measurements were performed using the HDL and LDL/VLDL Quantitation Kit from (catalogue # MAK045-1KT, Merck Sigma, Darmstadt, Germany) following the manufacturers protocol. Per manufacturers protocol, PPP samples were used to measure cholesterol parameters. All measurements were completed within 7 days of sample collection from fresh PPP maintained at 4 °C.

### Flow cytometry analysis of circulating EVs

PFP samples thawn from– 80 °C were analysed using the CytoFLEX S N2-V3-B5-R3 Flow Cytometer from (product # B78557, Beckmann Coulter, Brea, USA).

Measurements were carried out using the “slow” flow rate (10 µL/min) for 2 min and events were obtained from a previously optimized gate for EVs detection that was set up following a protocol outlined in [3, 4] using Flow Cytometry Sub-micron Particle Size Reference Kit (catalogue # F13839, Invitrogen, Carlsbad, USA) and shown in Supplementary Fig. 1. Antibodies were diluted 10-fold in 0.1 μm filtered Annexin Binding Buffer (ABB: 10 mmol HEPES, 140 mmol NaCl; 0.25 mmol CaCl_2_; pH: 7.4–7.5) and centrifuged at 12,600 g for 10 min at 4 °C, the supernatants were carefully transferred (ensuring that aggregated antibody pellets are not touched) to new labelled tubes.

For mice, 1 µL PFP and for humans, 4 µL PFP was used. These volumes were selected after preliminary optimization tests were performed with concentration curves ensuring that particle numbers did not saturate the detectors. With the selected concentration, sufficient particle numbers were present without causing a swarm effect. Each sample was placed into an Eppendorf tube along with antibody mixes shown in Supplementary Table 2. Tubes were protected from light and placed at 4 °C for 30 min. After the initial PFP antibody incubation, 30 µL count check beads from Sysmex (Order no: 05-4010 Lot: KW190620, 37920 particles/mL (± 10%)) were added and further 0.1 μm filtered ABB was added to reach a final volume of 300 µL.

Flow cytometry data were analysed using CytExpert2.1 (Beckman Coulter, Brea, USA) and exported to office365 Excel (Microsoft, Redmond, USA) for processing before analysis, using the following formula:

$$ \begin{aligned} & {\text{EV positive Events}} \\ & \quad = \left( {\frac{{{\text{fluorescent events detected}} - {\text{staining background}} }}{{{\text{bead count}}}}} \right) \\ &\qquad  \times{\text{bead concentration}}\times\left( {\frac{{{\text{EV dilution}}~}}{{{\text{bead dilution}}}}} \right)  \end{aligned}$$

### Bead-based analysis of human PFP-derived lEVs

The MACSPlex EV Kit IO, human (catalogue # 130-122209, Miltenyi Biotec, Bergisch Gladbach, Germany) was used to measure PFP from three patients from group A and from three patients from group B (for human samples). The MACSPlex EV Kit IO, mouse (catalogue # 130-122-211, Miltenyi Biotec, Bergisch Gladbach, Germany) was used for mice samples, PFP samples were pooled from 6 animals from each group (WT, PCSK9, and LDLR) after HFD. PFP was thawn from − 80 °C, and 1 mL from each human sample and 0.5 mL from pooled mouse samples were centrifuged for 40 min at 20,000 g at 4 °C. Supernatant was discarded and pellets were each re-suspended in 100 µL PBS, and re-centrifuged (40 min at 20,000 g at 4 °C). Supernatants were discarded, and pellets were each re-suspended in 100 µL PBS.

The total protein concentration of the pellet was determined by Micro BCA assay (catalogue # A55864 ThermoFisher, Waltham, Massachusetts, USA) and 10 µg total protein was used in each MACSPlex reaction. The “overnight” protocol was followed as specified in the manufacturer’s manual. As a negative control, a sample containing only MACSPlex Reaction Buffer, Detection antibody cocktail, and Capture Beads was used as a negative control. Measurements were performed using the CytoFLEX S N2-V3-B5-R3 Flow Cytometer (product # B78557, Beckmann Coulter, Brea, California, USA). Measurement was taken on medium speed (30 µL/minute). Gating strategy was made based on negative control sample and can be seen in Supplementary Fig. 2.

Each sample was analysed by subtracting the negative control signal from each marker with any negative values zeroed. For each sample, the isotype control with the highest signal was then subtracted from each marker. Averages were then calculated from each marker. All markers were then normalised to the average of standard EV markers (CD9, CD63, and CD81) and graphed in GraphPad Prism 8.2.0.

### Statistical analysis

Statistical analysis was performed using GraphPad Prism version 8.2.0 (GraphPad Software, Inc.). Values are presented as mean ± standard deviation with individual point plots. Shapiro-Wilk normality test was used to assess normality of all groups. For groups with normal distribution, unpaired student t test was performed and for groups that were non-normal, the non-parametric Mann-Whitney U test was applied. For comparisons of data sets within one group, a paired t test or a non-parametric Wilcoxon signed-rank test was selected depending on the normality of the samples.

## Introduction

With increased global aging, atherosclerotic cardiovascular disease (ACVD) has become the leading cause of deaths worldwide [5]. Primarily, fatalities occur from acute myocardial infarction (MI) [6, 7], with blockages to the carotid and cerebral arteries causing the first and second highest levels of stroke related fatalities respectively [8]. Low-density lipoprotein (LDL) and oxidised-LDL are key biomarkers in ACVD genesis and development, causing foam cell formation, macrophage recruitment and increasing fatty deposits on arterial walls [9]. Extracellular vesicles (EVs) began to gain attention in the last decade or more in all areas of biological research [10] due to their involvement in vast numbers of pathologies [11–13]. More recently, EVs have been shown to be involved not only in physiological but also in pathogenic processes both in vivo and in vitro, particularly in the field of oncology [13, 14] and CVD [11]. Lately, EVs have also shown strong potential as therapeutic agents for many diseases [15] including a variety of cardiovascular related health detriments [16, 17].

Modern “Western diets” based on high fat content and “fast foods” are widely noted for their contribution to dysfunction of cholesterol and lipoprotein metabolism leading to ACVD [18, 19]. They are shown to be among the forefront in cause of obesity, MI, stroke, and diabetes [19, 20] are even believed to play a crucial role in many other co-morbidities such as cancer [21]. Being that an increase in cholesterol has been historically noted as associated with poor diet [22, 23], our investigation set out using a high fat diet (HFD) in mice models to assess the cholesterol changes and how such alterations may affect circulating EV levels. Due to the increasing awareness in recent years of the impact genetics plays as a primary cause in cholesterol dysregulation [24, 25], we also used lipoprotein related genetically modified animal models to investigate EV alterations.

Our laboratory has demonstrated in 2016 [26] that LDL and EVs associate in blood plasma. Based on this, we hypothesized that EVs may also play a crucial role in atherosclerosis development, possibly affecting cellular internalisation of cholesterol in macrophages, foam cell formation, and endothelial interaction in the initial stage of arterial plaque build-up. The LDL receptor (LDLR) is involved in LDL clearance from blood circulation through guided cellular internalisation for degradation [27]. Considering this, we became interested in the roles of LDLR in regulating circulating EV levels and to study how this relates to LDL metabolism. Previously, LDLR gene silencing or knock-out (KO) strategies have been used extensively by other researchers to study the roles of this gene in influencing total cholesterol (TC) and LDL-cholesterol (LDL-C) levels [28, 29]. However, in this study, we have also opted to investigate the role LDLR plays in determining EV levels as well as, in cardiac function, and in atherosclerosis development in murine models.

Another important gene in ACVD is proprotein convertase subtilisin/kexin type 9 (PCSK9), a negative regulator of LDLR, which has become a key therapeutic target for the regulation of LDL-C in the most severe forms of clinical hypercholesterolaemia. Although much work is still ongoing in exploration of the PCSK9 biology, various inhibitors have been readily available and authorized for clinical use to help reduce LDL levels in chronic hypercholesterolaemic and ACVD patients for several years now [30], with FDA approval since 2015 [31]. Clinical trials are now in the planning stages, where CRISPR/Cas9 technology may be used in humans to KO the PCSK9 gene, specifically targeting hepatocytes [32]. Based on the significant interest in PCSK9 and its relationship with LDL levels, we also studied circulating EV levels in a PCSK9^−/−^ model.

Our principal aim in this study was to assess the levels of circulating EVs relative to the levels of cholesterol, primarily LDL-C. Furthermore, we evaluated hypo- and hypercholesterolaemic models in comparison to a normocholesterolaemic model in order to obtain additional insights into the relationship between circulating EV and cholesterol levels. For this reason, we have chosen to study gene KO mouse models. We studied LDLR^−/−^ mouse model where we could observe the changes occurring in an increased LDL-C environment and the PCSK9^−/−^ mouse model, where investigations could take place in a lowered cholesterol setting. Some studies show that hypocholesterolaemia can have a genetic contributing factor [33] while others show poor diet has a role in the increased cholesterol levels [19]. Although both may have their own role in increasing LDL levels, it is established that high circulating LDL-C is an indicator of poor cardiovascular prognosis in humans, with increased risk of atherosclerosis [34]. Therefore, this study also evaluates the circulating EV levels in human PFP from normal and hypercholesterolaemia patients to understand how EV levels may be relevant in clinical atherosclerosis and patient cardiovascular health.

## Results

### Cholesterol levels, and body mass measurements of mice at baseline, after HFD, and at old age

**Fig. 1:**
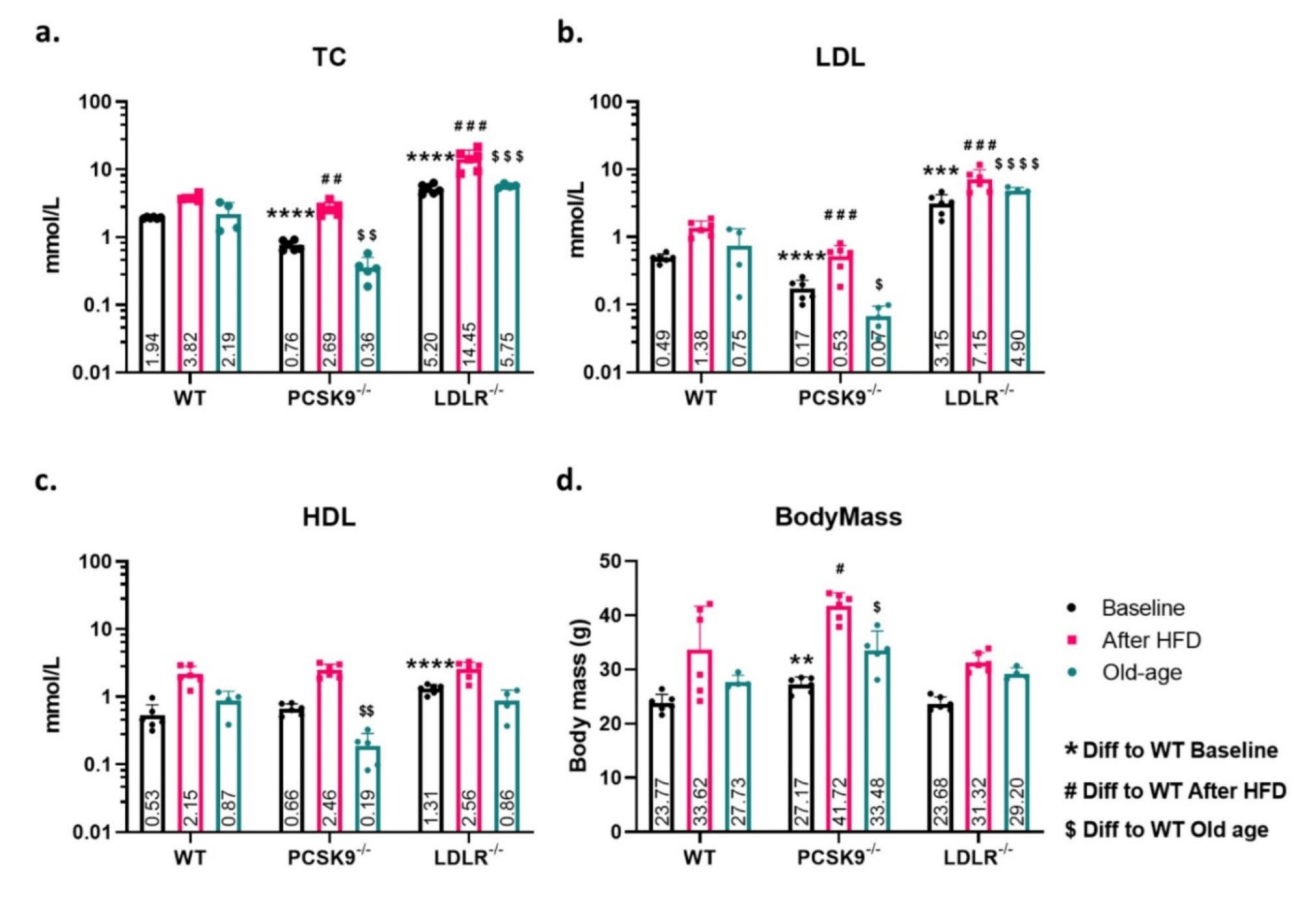
Cholesterol levels, body mass (BM) and fatty tissue observed in cholesterolaemia mice models in young, older, and high fat diet (HFD) mice. (**A**) Graphs showing, total cholesterol levels in PCSK9^−/−^ and LDLR^−/−^ mice compared to wild type (WT) mice at baseline, old age and after HFD. (**B**) A graph showing, low-density lipoprotein levels in PCSK9^−/−^ and LDLR^−/−^ mice compared to wild type (WT) mice at baseline, old age and after HFD. (**C**) A graph showing, high-density lipoprotein levels in PCSK9^−/−^ and LDLR^−/−^ mice compared to wild type (WT) mice at baseline, old age and after HFD. (**D**) A graph displaying the BM of all three mice groups at baseline, at old age, and after HFD

TC, LDL-C and HDL-C levels were initially measured in mice at 11 weeks of age before treatments (Baseline condition), after 12 weeks of HFD (HFD condition), or after reaching 22 months of age on normal diet (old age condition). TC levels in PCSK9^−/−^ mice were significantly lower than WT in all three conditions, whereas in LDLR^−/−^ mice serum cholesterol levels were significantly increased at all three conditions (Fig. 1A). LDL levels were significantly altered in both KO models, with the PCSK9^−/−^ mice showing a significant reduction in LDL levels in all conditions while the LDLR^−/−^ model shows a significant increase in all conditions (Fig. 1B). HDL levels were significantly lower in the PCSK9^−/−^ mice only in the aged animals and increased in the LDLR^−/−^ group only at baseline, however upon HFD feeding, there were no significant changes (Fig. 1C). Together these results confirm an opposing effect on cholesterol levels caused by the LDLR and PCSK9 genes. We also observed an overall direct correlation between the levels of all types of cholesterol measured. Individual readings of cholesterol from all mice and all time points can be found in can be found in Supplementary Table 3.

In addition to measuring cholesterol levels, body mass (BM) of mice were determined for all groups during the study. It was found that despite having improved TC and LDL-C levels, the PCSK9^−/−^ model had a statistically significant increase in BM (Fig. 1D) at baseline, HFD, and old age while WT or LDLR^−/−^ showed no significant difference. A visual increase in visceral fatty tissue in PCSK9^−/−^ mice can also be seen in Supplementary Fig. 3.

### Cardiovascular function and atherosclerotic plaque evaluation in mice at baseline, after HFD and old age

**Fig. 2:**
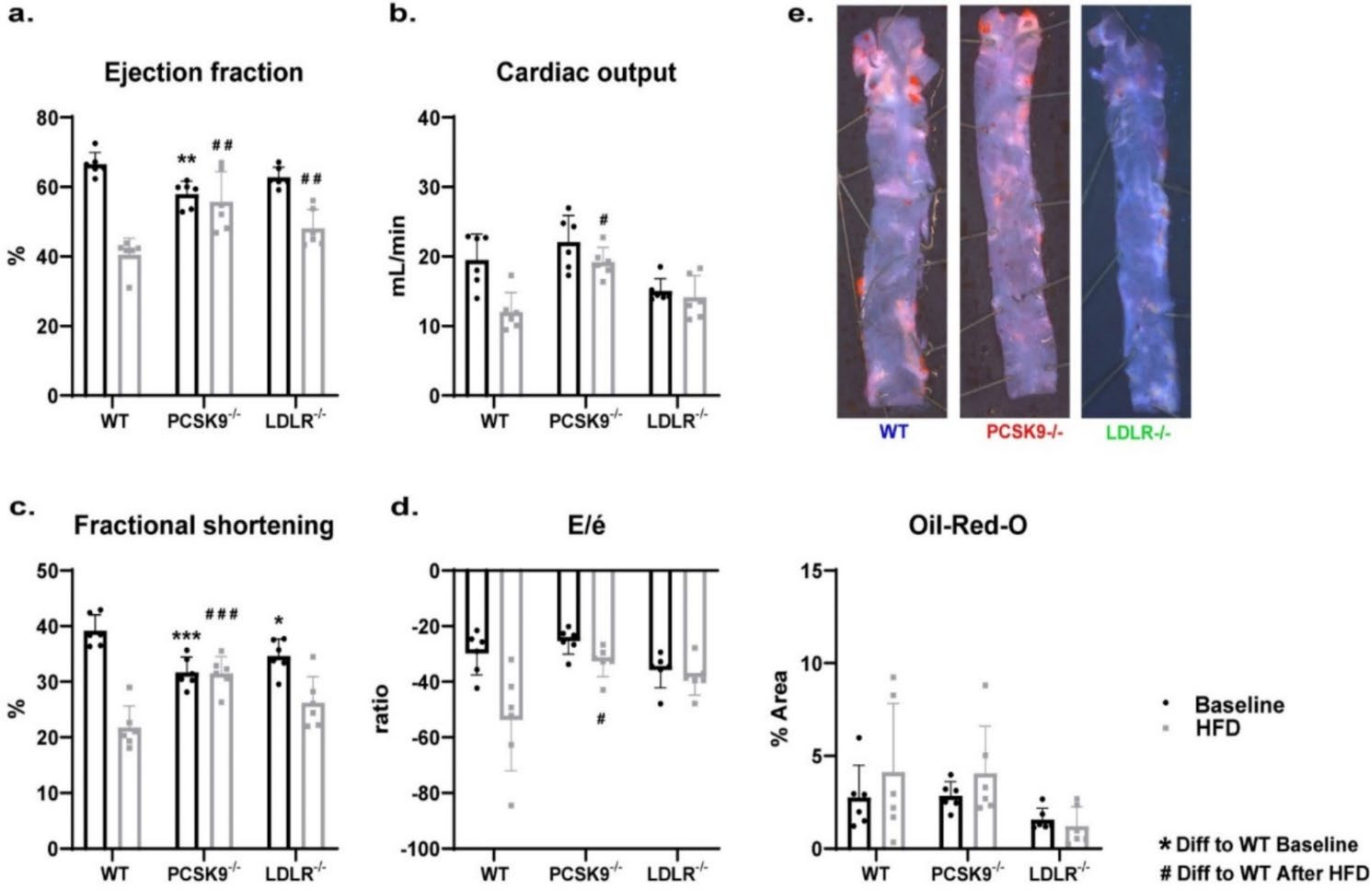
Four separate cardiovascular parameters measured in all mice at baseline and after high fat diet (HFD), and an analysis of atherosclerotic plaque was performed post-termination. (**A**) A graph showing the left ventricular ejection fraction of our knockout mice models compared to WT mice. (**B**) A graph showing the cardiovascular output of all mice models. (**C**) A graph displaying fractional shortening measurements of mice. (**D**) E/é ratio measurements taken HFD mice. (**E**) Oil-Red-O staining of the aortic arches shows quantifiably in a graph the plaques measured, as well as images showing one arch from each group (of HFD mice), as a representation of its group

Cardiovascular function and atherosclerotic plaque were assessed at baseline, after HFD, and at old age. Following HFD, echocardiography revealed that the cardiovascular function of PCSK9^−/−^ mice was statistically relatively retained compared to WT in all measured parameters (Fig. 2). Cardiovascular function was assessed by ejection fraction (Fig. 2A), cardiac output (Fig. 2B), fractional shortening (Fig. 2C), and the ratio of early diastolic mitral inward flow velocity to early diastolic mitral annulus velocity (E/e’), a parameter assessing left ventricular filling pressure (Fig. 2D). In contrast, aside for ejection fraction (Fig. 2A) the LDLR^−/−^ mice showed no difference in the other three cardiovascular function parameters compared to WT mice, (Fig. 2B–D). Following HFD and echocardiography, mice were terminated, and aortic arches were removed and isolated for atherosclerotic plaque evaluation after staining with Oil-Red-O. Despite the changes in cholesterol levels (Fig. 1) and cardiac function (Fig. 2A–D), we did not observe statistically significant changes in atherosclerotic plaques between WT, PCSK9^−/−^, and LDLR^−/−^ mice following HFD (Fig. 2E).

**Fig. 3:**
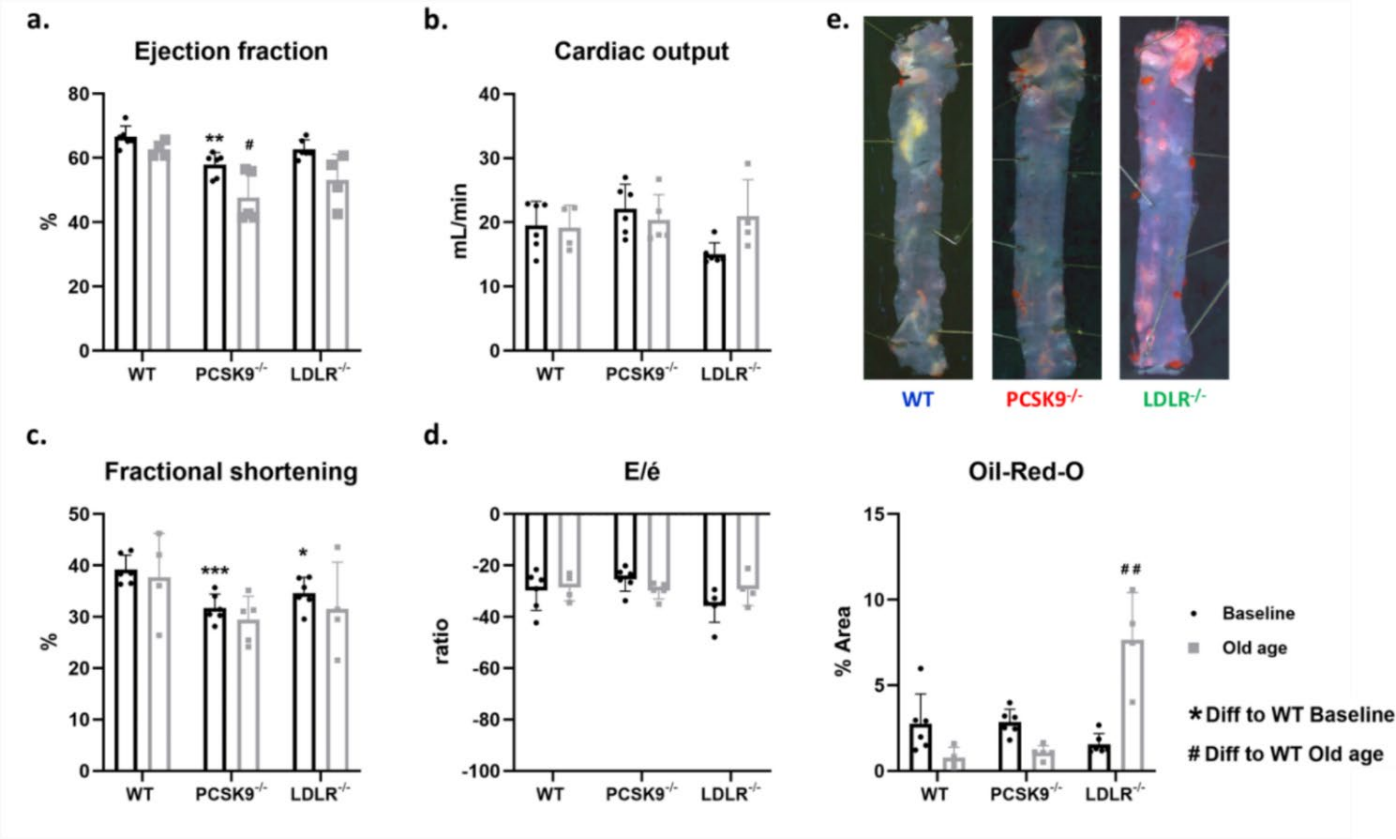
Four separate cardiovascular parameters measured in all mice which survived to old age, and an analysis of atherosclerotic plaque was performed post-termination. (**A**) A graph showing the left ventricular ejection fraction of our knockout mice models compared to WT mice. (**B**) A graph showing the cardiovascular output of mice model at old age. (**C**) A graph displaying fractional shortening measurements of mice at an older age. (**D**) E/é ratio measurements taken from old age mice. (**E**) Oil-Red-O staining of the aortic arches shows quantifiably in a graph the plaques measured, as well as images showing one arch from each group (of old aged mice) as a representation of its group

Cardiovascular function and atherosclerotic plaques were also measured at old age. With the exception of a decreased ejection fraction (Fig. 3A) in the PCSK9^−/−^ mice, all other cardiac function parameters were not statistically significantly changed compared to WT animals (Fig. 3B–D). Furthermore, we observed no difference in any of the cardiovascular function parameters between LDLR^−/−^ and WT (Fig. 3). However, as opposed to HFD, at old age we did observe a significant increase in atherosclerotic plaques measured in the LDLR^−/−^ group compared to WT mice, while PCSK9^−/−^ showed no difference to WT animals. Oil-Red-O stained aortic arches of mice before HFD can be seen in Supplementary Fig. 4.

Compared to baseline, HFD shows more effect on reduction of cardiovascular function as measured with ultrasonic doppler, and old age is where we start to observe plaques in mice. Furthermore, survivability was assessed and showed PCSK9^−/−^ had the most mice surviving to old age while LDLR^−/−^ showed the earliest deaths of group members (Supplementary Fig. 5). Additional cardiovascular function parameters were also assessed by echocardiography to determine wall thickness (Supplementary Fig. 6), revealing no significant changes amongst mice groups except when looking at LVPW; d and LVAW; d.

### Flow cytometry analysis of circulating large-sized EVs in mice at baseline, after HFD, and at old age using EV and lipoprotein markers

**Fig. 4:**
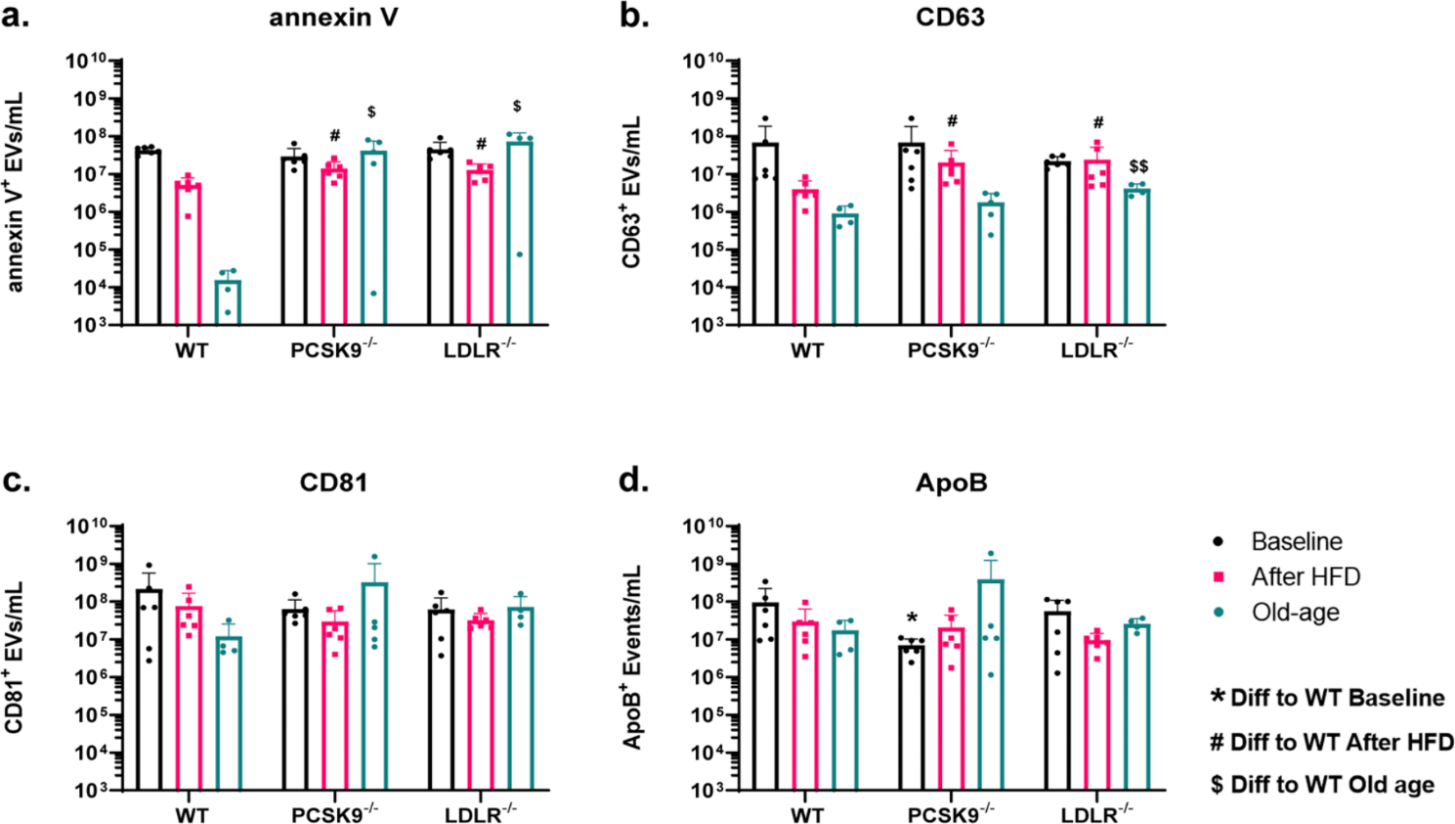
Large extracellular vesicle (lEV) level measured and categorised based on genetic backgrounds of mice as well as compared between baseline, after HFD, and at old age. (**A**) Based on annexin V staining in the lEV gate, comparison shown between wild type (WT) mice, PCSK9^−/−^, and LDLR^−/−^ at baseline, after HFD, and at old age. (**B**) Similarly, based on the CD63 antibody, both PCSK9^−/−^ and LDLR^−/−^ are compared to WT mice, at 11 week old baseline, after HFD, and mice at old age. (**C**) Shows the CD81^+^ measured in KO mice groups at baseline, after HFD, and at old age, in comparison to WT mice. (**D**) ApoB level measured between groups at baseline, after HFD, or at old age

Analysis of large-sized EV (lEV, vesicles with a diameter > 200 nm) levels was performed using flow cytometry with fluorescently conjugated antibodies and annexin V. Quantification of lEV sized annexin V^+^ particles showed significant changes in PCSK9^−/−^ and LDLR^−/−^ both after HFD as well as at old age, with no significant differences observed at baseline (Fig. 4A). Within the same gate, CD63^+^ particle levels showed a significant increase in both PCSK9^−/−^ and LDLR^−/−^ mice compared to WT after HFD. However, at old age. only the LDLR^−/−^ group showed a statistically significant decrease compared to WT (Fig. 4B). In contrast to annexin V and CD63, the CD81 marker showed no statistically significant change in any of the conditions (Fig. 4C). Additionally, ApoB measurements were taken, using the same EV gating strategy and revealed a statistically significant reduction in levels in PCSK9^−/−^ mice only at baseline, but not after HFD or at old age compared to WT (Fig. 4D). Further analysis was performed by combining all mouse models together, revealing a statistically significant decrease in annexin V^+^ lEV levels after HFD and statistically significant decrease in CD63^+^ lEV levels after HFD and at old age. However, no statistically significant changes in ApoB^+^ and CD81^+^ lEV levels was observed (Supplementary Fig. 7).

### Circulating EV and lipoprotein levels in normocholesterolaemic and hypercholesterolaemia patients

**Fig. 5:**
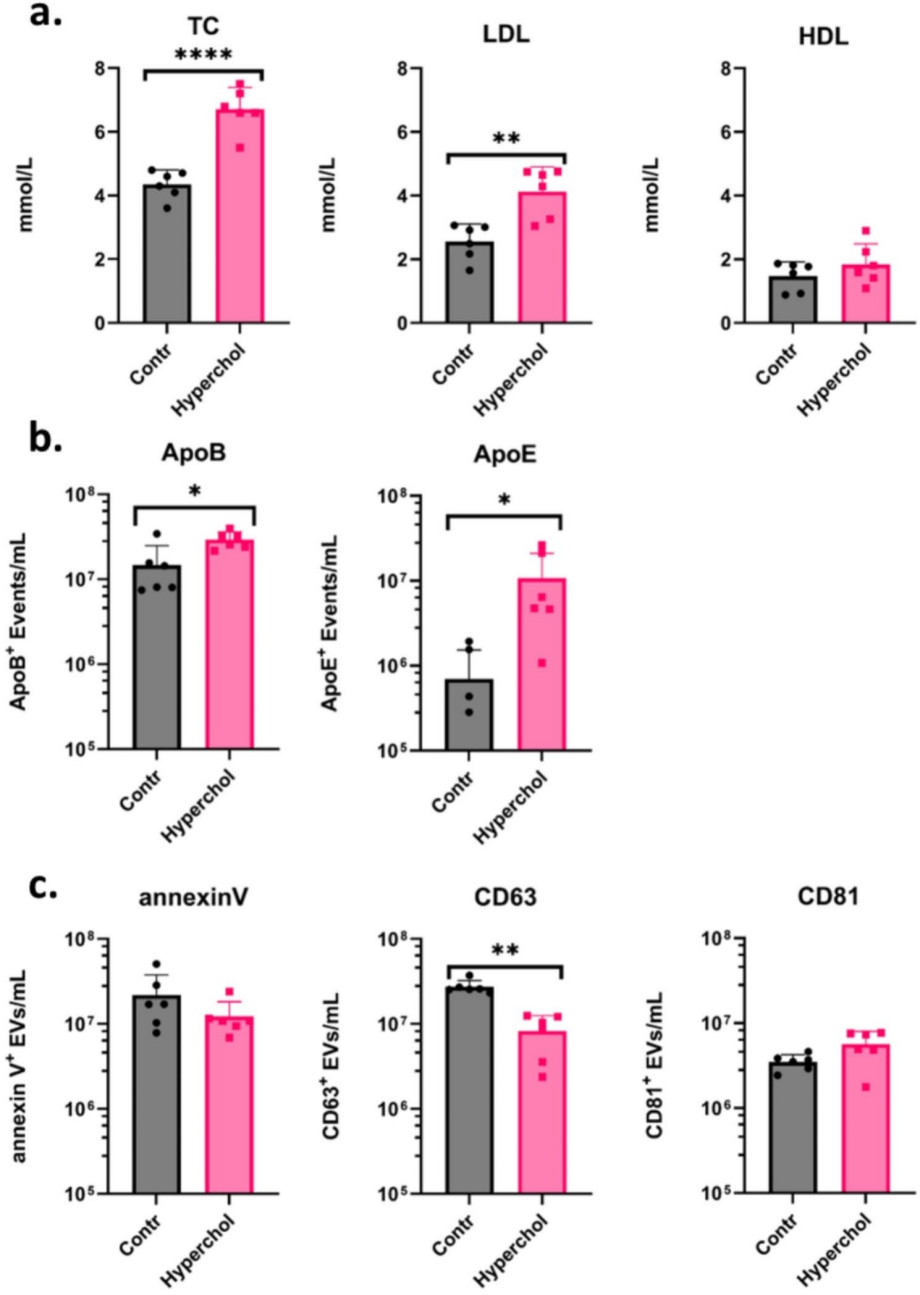
Patient cholesterol levels measurements of two groups of patients, hypercholesterolaemic group and normal cholesterol level group shown here. Flow cytometry measurements from platelet free blood plasma from patients were taken for, known extracellular vesicle, as well as lipoprotein markers. (**A**) Two groups of patients separated by total cholesterol (TC) levels. TC, low-density lipoprotein, and high-density lipoprotein levels are compared between the two groups. (**B**) Graphs showing both, circulating Apolipoprotein (Apo) B and ApoE in normal and hypercholesterolaemic groups. (**C**) Three graphs comparing, annexin V, CD63 and CD81 levels (respectively) in both patient groups

TC, LDL-C and HDL-C levels were measured in patients with no prior medical history of ACVD admitted to an emergency department with chest pain. Patients were divided into two groups: those with hypercholesterolaemia and those with normal cholesterol levels. We found that hypercholesterolaemic patients had significantly increased levels of circulating TC and LDL-C, while HDL-C was not significantly altered between the two groups (Fig. 5A). Similarly to the mouse models, CD63^+^ EV levels were significantly reduced in hypercholesterolaemia compared to normocholesterolaemic patients (Fig. 5B). However, no significant change was observed in case of annexin V^+^ and CD81^+^ EVs (Fig. 5B). Apolipoprotein levels were also measured within the lEV gate and revealed that both ApoB and ApoE were significantly elevated in the hypercholesterolaemic compared to normocholesterolaemic patients (Fig. 5C).

### Bead-based analysis of PFP-derived lEVs

**Fig. 6:**
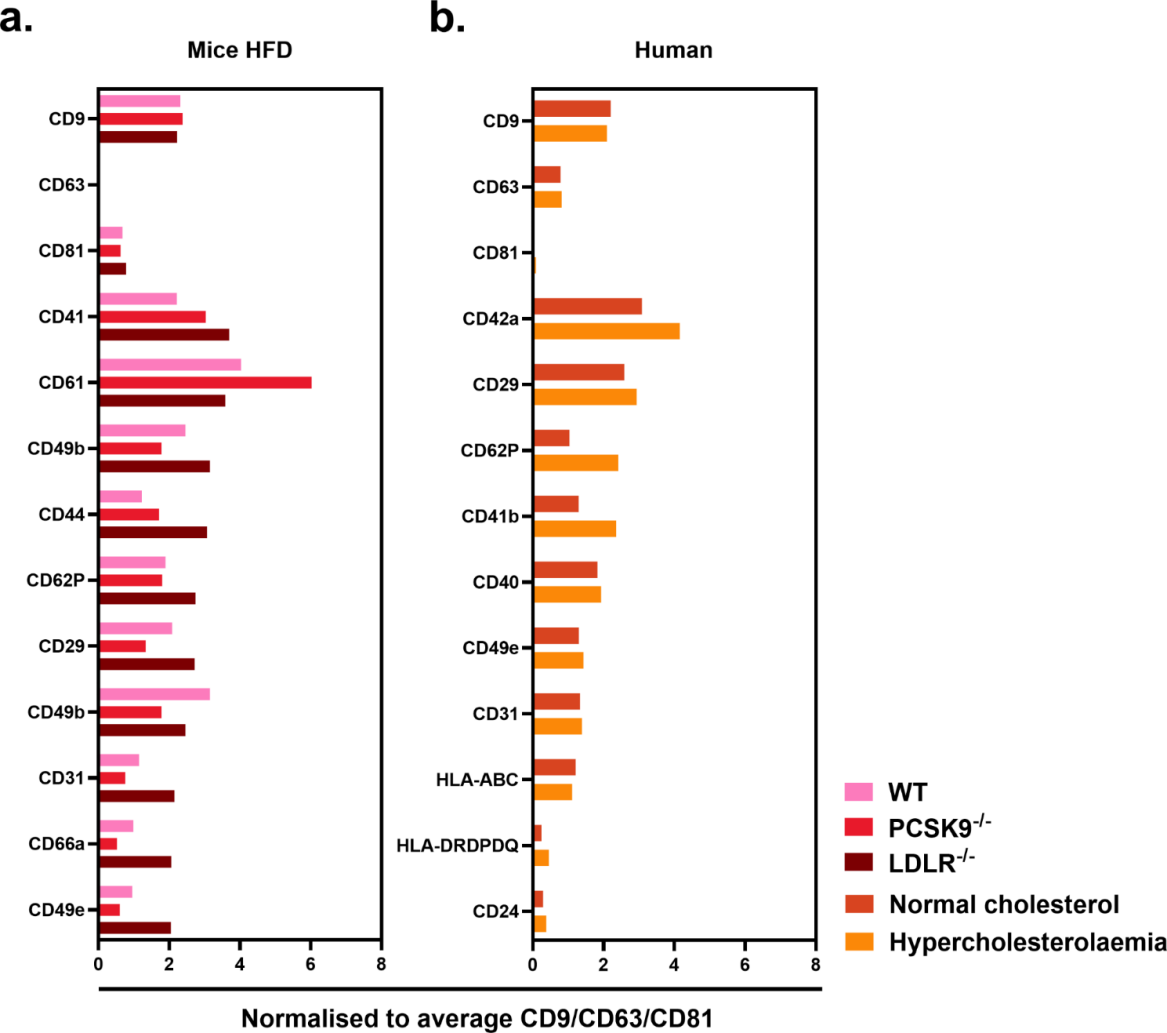
MACSPlex bead based exosome detection qualification and semi-quantification of potential extracellular vesicle and exosome sub-types. Here the Allophycocyanin (APC) mean fluorescence intensity (MFI) of markers were assessed and normalised to the average of the CD9, CD63, and CD81 markers. Samples above fold change 1 are considered to be of relative quantity in measured samples. (**A**) Graph showing the marker levels detected in wild type, PCSK9^−/−^, and LDLR^−/−^ mice after high fat diet. (**B**) Graph showing the marker levels detected in patients with normal and hypercholesterolaemia

The MACSPlex bead-based EV array was used to assess the levels of 37 markers as potential sources and cellular origin of the lEVs present in PFP samples. In the case of both normal and hypercholesterolaemia patients (Fig. 6A), and for HFD mice (Fig. 6B), we analysed the top 10 most prominent markers detected with the bead-based array. For human samples, we found elevated positivity in hypercholesterolaemic samples compared to normocholesterolaemic samples for two common platelet markers CD41b, CD42b, as well as CD62P, a marker of endothelial cells and activated platelets. Other markers found to be present at similarly high levels for all human samples were CD9, CD24, CD29, CD31, CD40, CD49e, HLA-ABC, and HLA-DRDPDQ.

With HFD mice groups (Fig. 6B), we found markers decreased in PCSK9^−/−^ and elevated in LDLR^−/−^ compared to WT mice, following the same changes observed in cholesterol levels. These included CD29, CD31, CD49b, CD49e, and CD66a. As opposed to these markers, we found CD61 to inversly follow cholesterol levels. Other markers were found elevated for both PCSK9^−/−^ and LDLR^−/−^ compared to WT mice, including CD40, CD41 and CD44. Lastly, markers found to be present at similar high levels for all mouse models were CD9, CD62P and CD81.

For both human samples and HFD mice, we also showed the less abundant markers detected using a bead-based kit. This additional data can be found in Supplementary Fig. 8. Based on abundance and patterns displayed by certain markers in relation to cholesterol, particularly of interest, were the CD29 and CD62P markers. We decided to assess single lEV levels using the aforementioned markers. Supplementary Fig. 9 shows the single lEV measurements taken from both (Supplementary Fig. 9A) clinical samples and (Supplementary Fig. 9B) the mouse models. As seen in the graphs, no significance was observed in either humans or mice models with regards to the CD29 or the CD62P markers.

Finally, we assessed the colocalization of EVs with lipoproteins. To investigate this, we used Single-particle interferometric reflectance imaging sensor technology to capture exosomes on an immuno-surface capture chip containing spots of anti-CD63, -CD9, and -CD81 capture antibodies. Here the Leprechaun (instrument # 50TS8VNANO, Unchained Labs, Pleasanton, USA) was used with the Leprechaun exosome human Tetraspanin kit (catalogue # 251–1044). Following capture onto the chip, human PFP samples were stained with a cocktail of fluorescently labelled anti-CD9, -CD63, and -ApoB antibodies. We found that ApoB staining did not colocalize with EVs from human PFP captured by anti-CD63, -CD9, and -CD81 antibodies and that overall lower EV concentrations were found in hypercholesterolaemic patients (Supplementary Fig. 10).

## Discussion

Cholesterol levels, primarily LDL-C, are currently the most common biomarkers for ACVD [35] development as well as prognostic outcome. Our work shows that HFD contributes to elevated LDL-C level in our mouse models studied compared to both young mice at baseline and mice at old age. In regard to genetic background, as expected, we observed an opposing effect of PCSK9^−/−^ and LDLR^−/−^ in decreased or increased LDL-C and TC levels, respectively, compared to WT mice at all stages, including, baseline, after HFD, and at old age. After HFD, we expected increased cholesterol and indeed the TC and LDL-C levels were increased after HFD, however, we observed the genetic background of mice having a greater impact on cholesterol levels. Interestingly, we observed that HDL-C levels, sometimes referred to as “good cholesterol” [36] also rises after HFD. Moreover, HDL-C levels in LDLR^−/−^ mice showed a highly significant increase over the WT mice already at baseline before commencing HFD. Due to the fact that HDL is involved in the removal of LDL and very low-density lipoproteins (VLDL) by transporting these lipoproteins to the liver for degradation and storage, this data might suggest that in the absence of the LDLR gene, HDL production is upregulated in order to assist LDL removal thereby aiding in maintaining cholesterol homeostasis. It has also recently been suggested that increased HDL levels may actually not be as beneficial as once thought [37, 38], and perhaps the increased HDL levels may be contributing to increased lipidomic instability and dysfunction. However, it is important to note that the regulation of lipoproteins and their concentrations in mice differs compared to humans [39].

An important observation from our data is the increased body mass and visceral fatty tissue seen in the PCSK9^−/−^ mice, which has also been shown in an earlier study [40]. The PCSK9 gene serves many functions in the body aside from cycling and degradation of the LDLR. Many consider PCSK9 a key player and therapeutic target in cardiovascular pathology [30, 41], however, disruption of normal PCSK9 function may also lead to other health detriments [42]. For one thing, PCSK9 is a regulator of CD36. With PCSK9 deficiency or in the PCSK9^−/−^ animal model studied here, CD36 expression may be increased. CD36 in turn is highly involved in the transport of triglycerides as well as long-chain fatty acids [43]. With lower levels, or in the absence of PCSK9, an increase in CD36 could be one explanation for accumulation of lipids in various tissues [43, 44].

Our data also show that after HFD, PCSK9^−/−^ mice had almost completely retained cardiovascular function with higher ejection fraction, cardiovascular output, fractional shortening, as well as a prognostically favourable E/e’ ratios compared to thier WT counterparts. This highlights a cardioprotective function of PCSK9 suppression in HFD, which conforms with the use of PCSK9 inhibitors such as alirocumab (Praluent) and evolocumab (Repatha) for treatment of patients with hypercholesterolaemia. Despite the potential cholesterol lowering effect of PCSK9 inhibition, higher body mass has also been observed in patients with PCSK9 mutations, which raises questions as to how gene editing of PCSK9 may impact the long-term health and well-being of patients [40, 45].

Due to the fact that ACVD is the most common cause of CVD, analysis of atherosclerotic plaque burden may be considered a more important assessment of cardiovascular health. In mice, the most common place to find arterial plaque formation is in the aortic root [2]. For this reason, we opted to analyse the atherosclerotic plaque built up on the aortic arch of our mice groups, where we can compare the likelihood of arterial blockages in these cholesterolaemic models. Here we found no significant difference between PCSK9^−/−^, or LDLR^−/−^ at baseline or after HFD (compared with WT animals). With an average lifespan of approximately 2 years, BL/6 background mice can make an ideal model for age related research [46]. With aging, CVD risk increases [7, 47] and as such we decided to include an old age condition into our research. We found that old mice in the LDLR^−/−^ group had a significant increase in plaques compared to WT mice. This finding suggests that, at least in short term studied here (12 weeks), HFD may not have a strong effect on plaque formation compared to a long term exposure to high cholesterol levels, as seen with LDLR^−/−^ at old age. This data may be more representative of what is seen with clinical investigations of hypocholesterolaemia and aging [7]. Indeed, if we consider that the cholesterol levels at baseline and old age are similar for WT and PCSK9^−/−^, unlike the elevated levels observed for LDLR^−/−^, it may explain why their plaque levels are significantly lower.

One of the key motivations to carry out this study, was to understand the relationship between levels of EVs and lipoproteins (primarily LDL), which was assessed by flow cytometry using the major EV and apolipoprotein markers. Compared to WT mice, only PCSK9^−/−^ mice had a significant decreased in ApoB at baseline. For annexin V and CD63, both LDLR^−/−^ and PCSK9^−/−^ had statistically significant increase after HFD and at old age. This was particularly interesting, as the cholesterol levels of both models were inversely related but their annexin V and CD63 positive EV levels were elevated. We have also observed that after HFD, annexin V, CD63 and, CD81 all have lower levels within each mouse model. This trend was supported when we combined all mouse groups and saw a significant decrease in annexin V and CD63 after HFD. This data suggests an inverse relationship between lipoproteins (primarily LDL) and EVs in mice. One explanation to this could be, that due to an increased level of lipoprotein, interactions between lipoprotein and annexin V and CD63 positive EVs in mice are more frequent, leading to increased cellular uptake of the EV-lipoprotein complexes through LDLR. It is possible that EVs may “piggyback” on the LDL for uptake, thus reducing EV levels in a lipoprotein rich environment. While HFD resulted in higher lipoprotein and lower EV levels on average, it was interesting that the LDLR^−/−^ group after HFD and at old age had similar EV levels to the PCSK9^−/−^ group despite having higher cholesterol levels. One explanation for this could be the possibility of other lipoprotein receptors such as Lectin-like Oxidized Low-Density Lipoprotein Receptor-1 (LOX-1), Scavenger Receptor Class B Type 1 (SCARB-1), or Lipoprotein Receptor-related Protein-1 (LRP1) partially replacing LDLR as a mediator of EV-lipoprotein complex uptake in LDLR^−/−^ mice.

Interestingly, data from human samples recapitulated the mouse models. We also observed that EV levels followed a similar decrease as seen in mice with high cholesterol and lipoprotein levels. Our data show that CD63^+^ circulating EVs were statistically significantly decreased in hypercholesterolaemia patients compared to normocholesterolaemic patients. However, decreased annexin V^+^ lEV levels in hypercholesterolaemic patients were not found to be statistically significant as observed in mice models. Taken together, these results are consistent with our hypothesis of a relationship between EVs and cholesterol.

While we see similarities between the EV and cholesterol levels measured in mice and humans, the developmental landscape of atherosclerosis is somewhat different between these species. MI incidences in humans stems primarily from plaque build-up in the coronary artery, while mice more often develop plaques in the root of the aortic arch [2] making MI incidence rare. Based on this and other distinctions between mouse models and human patients [48], it is difficult to speculate without further investigations as to the direct role of EVs in human ACVD development. We do know that inflammation is indicative of ACVD and can also exacerbate the condition [49], and therefore lEVs from patients were isolated in order to test their pro-inflammatory effects on human umbilical vein endothelial cells (HUVECs). As seen in Supplementary Fig. 11, some increase in gene expression of CD36 and PPARg has occurred only in HUVECs incubated with lEVs isolated from hypercholesterolaemia patients. It can also be seen that there is a reduction in VEGFA in HUVECs incubated with lEVs isolated from patients with hypercholesterolaemia. CD36 upregulation could be a sign of inflammation in both adipocytes and macrophages [50]. However, PPARg has been also linked to anti-inflammatory responses such as inhibiting TNFa [51] and VEGFA is also implicated in inflammation [52] and respectively we see an up- (PPARg) or down- (VEGFA) regulation in cells incubated with the same EVs.

We have already established however, that EVs and LDL associate in blood plasma [26] and it is also becoming widely accepted that EVs carry a protein corona [53] within a protein rich environment (such as blood plasma). Based on our findings, it is possible to speculate that EVs may be more readily cleared from circulation if associated with lipoprotein particles. Possibly, in individuals with high levels of circulating cholesterol, these LDL-EV associations will increase. Another hypothesis to explain this phenomenon may be due to reduced platelet or monocyte cellular activity in disease state, leading to reduced EV production and secretion. A recent paper has even shown numerous changes to the structure of EVs and protein interaction with EVs upon their release from cardiomyocytes after high cholesterol treatments in rats [54].

It has long been known that fasting versus prandial blood cholesterol levels are different [55, 56] in both healthy and diseased state patients. In our supplementary data (Supplementary Fig. 12), we show that CD63^+^ lEVs do not change significantly in fasting versus prandial states in young mice, where cholesterol levels do. This may further support that CD63^+^ EVs levels could assist clinicians in delivering a more accurate diagnosis to hypercholesterolaemia and overall cardiovascular health of patients.

It has previously been shown that platelet makers CD41b, CD42a, and CD62P are highly present in sEVs of human PFP [57]. Here, we show similar findings in lEVs from human and mice PFP. In contrast to the classic EV markers (CD9, CD63 and CD81), we found other markers to be more bound to EV-capturing beads in PFP of both mice and humans. CD29, CD42a, CD49e, and CD62P show positive correlations while CD61 inversely tracked the cholesterol levels.

One question that can be answered based on these findings, is that reduced EVs, in particular CD63^+^ lEVs, can give a clinical guidance as to a patient’s cholesterol regulation. Elevated LDL-C may not always cause cardiovascular diseases, and based on our data in supplementary Fig. 10, EV levels can be investigated independently of certain lipoproteins. Thus, further investigations into EV profiles may lead to better understanding of cardiovascular health and in future may allow for more precise prognostics and patient outcomes in those suffering with upregulated cholesterol.

## Conclusions

This study shows for the first time, an inverse relationship between LDL-C and EVs (specifically CD63^+^ lEVs). With two mouse models, we highlight PCSK9 and LDLR as genetic factors for development of CVD. We also highlight the relationship EV levels (in addition to cholesterol) have to atherogenesis. Finally, we also bring further evidence to the obesogenic properties of deficiency or KO of the PCSK9 gene, which may lead to other health detriments, notably diabetes, which need to be considered in the context of PCSK9 inhibition medication or future gene editing possibilities.

## Electronic supplementary material

Below is the link to the electronic supplementary material.

Supplementary Material 1

## Data Availability

No datasets were generated or analysed during the current study.

## Abbreviations

ABB: Annexin binding buffer
ACVD: Atherosclerotic cardiovascular disease
ApoB: Apolipoprotein B
ApoE: Apolipoprotein E
BM: Body mass
CVD: Cardiovascular disease
E/e’ ratio: Ratio of early diastolic mitral inward flow velocity to early diastolic mitral annulus velocity
EV: Extracellular vesicles
HDL: High-density lipoprotein
HDL-C: High-density lipoprotein cholesterol
HFD: High fat diet
lEV: Large extracellular vesicles
LDL: Low-density lipoprotein
LDLR: Low-density lipoprotein receptor
LDL-C: Low-density lipoprotein cholesterol
LOX-1: Lectin-like oxidized low-density lipoprotein receptor-1
LRP1: Lipoprotein receptor-related protein-1
LVRi: Left ventricular remodelling index
MI: Myocardial infarction
PCSK9: Proprotein convertase subtilisin/kexin type 9
PFP: Platelet free plasma
PPP: Platelet poor plasma
RWT: Relative wall thickness
SCARB-1: Scavenger receptor class B type 1
TC: Total cholesterol
VLDL: Very low-density lipoproteins
WT: Wild type
