# Supplementary material for: Reduced circulating CD63+ extracellular vesicle levels associate with atherosclerosis in hypercholesterolaemic mice and humans
Source: Cardiovasc Diabetol. 2024 Oct 17;23:368. doi: 10.1186/s12933-024-02459-w (PMC11487797; doi:10.1186/s12933-024-02459-w)
Supplement: Supplementary file 1 — Supplementary Material 1 [file 12933_2024_2459_MOESM1_ESM.docx]

**Supplementary Data: Reduced circulating CD63^+^ extracellular vesicle levels associate with atherosclerosis in hypercholesteraemic mice and humans**

**Methods**

**Tissue culture**

The isolation of human umbilical vein endothelial cells (HUVECs) is covered by the Ethical Committee Vote 200 (2005) from the State of Upper Austria. HUVECs were thawn from nitrogen and passaged into a tissue culture treated flask with endothelial cell growth media-2 (EGM2) + 10 % foetal bovine serum and allowed to grow to 80 % confluency. Cells were then detached using TrypLE™ Select Enzyme (catalogue # 12563011 Invitrogen, Carlsbad, USA) and centrifuged at 200 g for 4 minutes, the supernatant was discarded, and the cells resuspended in EGM2 (serum free). The cells were then plated into wells on a 6-well plate, each well containing 1 mL EGM2 (serum free). The cells were then left to grow until reaching a confluency of 70 % before beginning the gene expression assay. EGM2 media was removed from the wells, each well was washed with 1 x phosphate-buffered saline (PBS).

**IEV isolation and quantification**

1 mL of PFP from both normo- and from hypercholesteraemic patients was thaw from -80 ^o^C and centrifuged at 20,000 g for 40 minutes using refrigeration of 4 ^o^C. supernatants were discarded and resuspended in 100 µL EGM2 media and re-centrifuged at 20,000 g for 40 minutes using a refrigeration of 4 ^o^C. The supernatant was discarded, and the pellets were reconstituted in 105 µL EGM2 media. 5 µL of each sample was used to quantify the EVs using NTA.

Particle size and concentration was measured using a ZetaView Z-NTA instrument (Particle Metrix, Munich, Germany). Samples were diluted either 1:2,000 (isolates from hypercholesteraemic patients) or 1:250 (isolates from normocholesteraemic patients) using 1 x PBS. Across the flow cell, 11 positions were scanned, with each position measured twice as technical replicates. The instrument maintained 25 °C to retain a consistent Brownian motion. Measurements were attained ensuring 150-300 particles/frame. The sensitivity of the camera was set to 75 using a shutter speed of 80 FPS. The analysis parameters included: a maximum area: 1000, a minimum area: 5, with a minimum particle brightness: 20.

**Gene expression assay**

Based on the NTA measurements, 1.40E+09 lEVs were used per well in the wells where EVs were added in the 6-well plate. As a positive control, one well contained 10 µg/mL lipopolysaccharide (LPS) was used to induce inflammation. As a negative control, one well contained HUVECs and EGM2 media alone. Cells were incubated for 24-hours in a 5 % CO^2^ incubator at 37 ^o^C with 90%–95% humidity. After 24-hours, media was removed from wells and wells were washed with 1 mL 1 x PBS. PBS was removed and cells were suspended in 700 µgL of QIAzol lysis reagent (catalogue # 79306, Qiagen, Hilden, Germany). 140 µL chloroform was mixed into the samples for phase separation and samples were centrifuged at 20,000 g for 20 minutes maintaining 4 ^o^C refrigeration of samples. The upper clear phase was then pipetted onto columns from the GeneAid total RNA isolation kit (catalogue # RSD300, Geneaid, Biotech Ltd, New Taipei City, Taiwan) and steps for clean-up, washing and elution of samples were followed from the kit.

RNA was reverse-transcribed to cDNA using the SensiFAST cDNA kit (catalogue # BIO-65054, Bioline, London, UK) following the manufactures protocol. A qPCR of the cDNA was performed to determine gene expression of HUVECs incubated with lEVs isolated from either normocholesteraemic or hypercholesteraemic patients as well as HUVECs incubated with LPS (comparing them to HUVE cells alone). TaqMan probes for gene expression (catalogue # 4331182, Applied Biosystems, Waltham, Massachusetts, USA) were used, all containing a FAM fluorophore. The qPCR 2 x TaqMan™ Fast Advanced Master Mix (catalogue # 4444557, Applied Biosystems, Waltham, Massachusetts, USA) following the manufacturers protocol. Each well on the 384-well qPCR plate contained an HPRT1 (catalogue # 4448484, Applied Biosystems, Waltham, Massachusetts, USA) probe as an internal (housekeeping gene) control, this probe contains a VIC fluorophore.

The expression levels of the following genes were analysed for all biological conditions; CD36, CXCL1, HMOX1, IL6, PPARg, TRIB1, VEGFA.

The qPCR was run using the BioRad CFX Opus 384 Real-Time PCR System (BioRad C1000 Touch Thermal Cycler, Hercules, CA, United States).

**Single-particle interferometric reflectance imaging sensor (SP-IRIS)**

SP-IRIS measurements were performed on a Leprechaun instrument (Unchained Labs, Pleasanton, California, USA). Leprechaun Exosome human Tetraspanin capture kit (catalogue # 251-1044) was prepared in accordance with the manufacturer's protocol. PFP was diluted 1:5 in a 1x solution of the kit’s Incubation solution. Immunocapture of CD63, CD9, and CD81 EVs was performed using the Leprechaun Exosome human Tetraspanin capture kit. EV samples were incubated on background-scanned chips and analysed using Leprechaun instrumentation in both interferometric microscopy (IM) and fluorescence channels. Data was processed using Leprechaun Analysis 1.1 software to assess particle concentration and sub-population characteristics. Concentration of antibodies were left as manufacturer’s recommended, 0.6 µL/antibody/sample. The kit’s anti-CD9 and -CD63 detection antibodies were used, and the supplied anti-CD81 was substituted for an anti-ApoB conjugated FITC (catalogue # TR78654-02, Tresars, London, UK).

| **Group** | **No.** | **Anthropometric data** | | **Time of recruitment** | **Lipid profile** | | | | **Other lab data** | | | | | | |
| --- | --- | --- | --- | --- | --- | --- | --- | --- | --- | --- | --- | --- | --- | --- | --- |
|  |  |  |  |  | **Total chol.** | **LDL** | **HDL** | **TG** | **Glucose** | **HbA1C** | **eGFR** | **GOT** | **GPT** | **INR** | **CRP** |
|  |  | **Sex** | **Age** |  |  |  |  |  |  |  |  |  |  |  |  |
| **Normocholesterolaemia** | 7 | M | 39 | 01/02/2022 | 4.7 | 3.06 | 0.92 | 1.51 | 7.4 | - | 90 | 33 | 58 | 1.12 | 4 |
|  | 27 | M | 24 | 07/02/2022 | 4.8 | 2.92 | 1.77 | 0.2 | 5.6 | 5.2 | 90 | 25 | 25 | 1.03 | 0.7 |
|  | 28 | M | 35 | 07/02/2022 | 4.3 | 2.49 | 1.56 | 1 | 5.5 | 5.3 | 90 | 23 | 18 | 0.98 | 0.2 |
|  | 34 | M | 25 | 14/02/2022 | 4.1 | 2.17 | 1.86 | 0.7 | 5.3 | - | 90 | 21 | 12 | 0.97 | 0.4 |
|  | 41 | M | 58 | 17/02/2022 | 3.6 | 1.65 | 1.81 | 0.7 | 6.4 | - | 82.6 | 24 | 25 | 1.04 | 0.6 |
|  | 69 | M | 63 | 29/03/2022 | 4.6 | 3.01 | 0.88 | 3.1 | 5.5 | 5.7 | 90 | 22 | 18 | 1.15 | 0.6 |
|  |  |  |  |  |  |  |  |  |  |  |  |  |  |  |  |
| **Hypercholesterolaemia** | 13 | M | 39 | 01/02/2022 | 6.6 | 3.26 | 2.9 | 1.2 | 5 | 5.1 | 90 | 35 | 22 | 0.96 | 0.4 |
|  | 15 | M | 32 | 01/02/2022 | 5.5 | 3.04 | 1.8 | 1.45 | 4.6 | 5.2 | 87.7 | 27 | 27 | 1.07 | 0.1 |
|  | 24 | M | 48 | 04/02/2022 | 6.6 | 4.65 | 1.09 | 2.8 | 5.2 | - | 81.8 | 37 | 45 | 1.04 | 14.3 |
|  | 30 | M | 62 | 27/02/2022 | 7.2 | 4.76 | 1.59 | 1.6 | N/A | 5.5 | 5.1 | 50 | 100 | 0.93 | 1.9 |
|  | 48 | M | 38 | 01/03/2022 | 7.5 | 4.29 | 2.23 | 3.7 | 5.1 | 4.9 | 90 | 19 | 15 | 1.03 | 0.7 |
|  | 67 | M | 52 | 29/03/2022 | 6.8 | 4.75 | 1.41 | 1.6 | 10.2 | 6.6 | 88 | 44 | 74 | 0.93 | 4 |
| **Unpaired t-test  p value** |  |  | 0.59 |  | 3.6873E-05 | 0.0024 | 0.2768 | 0.169 | 0.946542 | 0.8902 | 0.304 | 0.0549 | 0.1942 | 0.187 | 0.3058 |
| **Significantly different?** |  |  | no |  | yes | yes | no | no | no | no | no | no | no | no | no |

**Supplementary Table 1.** A table showing parameters measured of each individual patient. All measurements presented in this table were taken by the Semmelweis Városmajor emergency clinic.

**
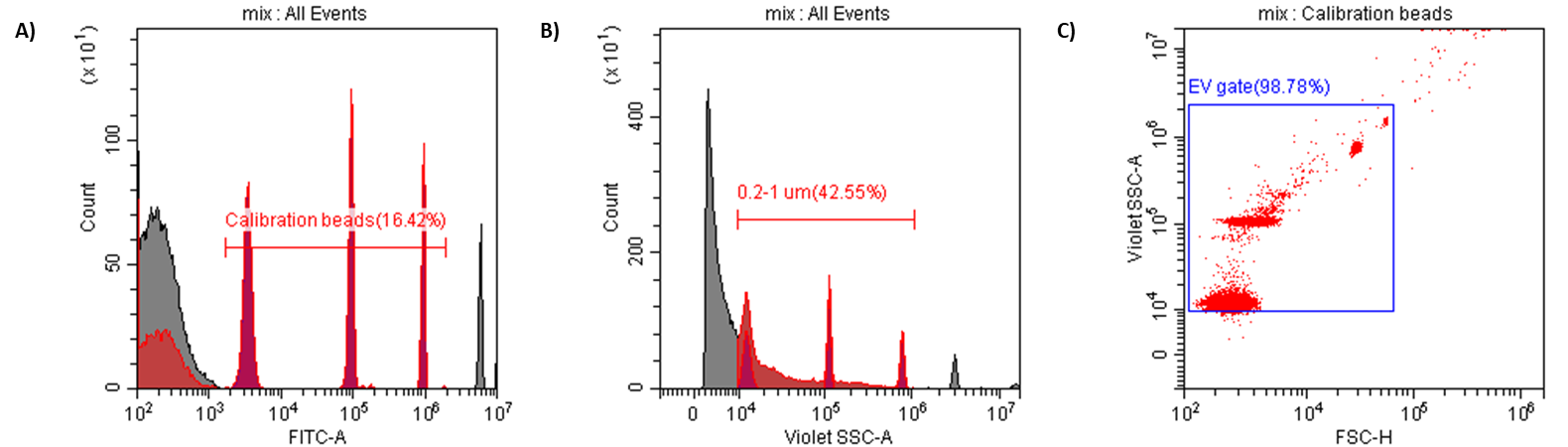
 Supplementary Figure 1.** EV detection gating based on the Flow Cytometry Sub-micron Particle Size Reference Kit provided by Invitrogen. (A) A graph shows the histogram of Count/FITC-Area, where (calibration bead) gating spans 0.2 um, 0.5 um and 1 um sized peaks. (B) A graph showing count/Violet SSC-area of beads – with a gating between the 0.2 – 1 um peaks. (C) A scatter plot using Volet SSC-area/FSC-hight, plotting the “Calibration beads” gate in a forwards/side scatter, showing the three populations of beads, around which the EV gate was set.

| **Species** | **Antibody** | **Provider** | **Catalogue No.** | **Conjugate** | **Volume (µL)** | **Cocktail No.** |
| --- | --- | --- | --- | --- | --- | --- |
| **Mice antibodies** | Annexin V | Invitrogen | R37175 | AlexaFlora647 | 7.2 | 1 |
|  | CD63 | Sony Biotechnology | RT1319520 | PE | 1.8 |  |
|  | CD81 | Sony Biotechnology | RT1124545 | APC | 1.2 | 2 |
|  | ApoB | Tresars | TR78654-02 | FITC | 1.5 |  |
|  | ApoE | Novus Biologicals | NB110-60531PCP | PerCP | N/A | N/A |
|  | CD29 | Sony Biotechnology | RT1111120 | Pacific blue | 1.5 | 3 |
|  | CD62P | Sony Biotechnology | RT1341520 | APC | 1.5 | 4 |
|  |  |  |  |  |  |  |
| **Human antibodies** | Annexin V | Invitrogen | R37175 | AlexaFlora647 | 7.2 | 1 |
|  | CD63 | Sony Biotechnology | RT2365095 | PerCP-Cy5.5 | 3.04 | 2 |
|  | CD81 | Sony Biotechnology | RT2347575 | Pacific blue | 1 | 3 |
|  | ApoB | Tresars | TR78654-02 | FITC | 1.5 |  |
|  | ApoE | Novus Biologicals | NB110-60531PCP | PerCP | 0.5 | 4 |
|  | CD29 | Sony Biotechnology | RT2115040 | APC | 1.5 | 5 |
|  | CD62P | Sony Biotechnology | RT2124520 | FITC | 2 |  |

**Supplementary Table 2.** The table shows antibody mixes used for flow cytometry. The table shows the specie of antibody, the antibody, the provider/vender, the catalogue number, the antibody conjugate, the volume of each antibody used per sample, and any cocktail mixtures used in simultaneous measurements.

**
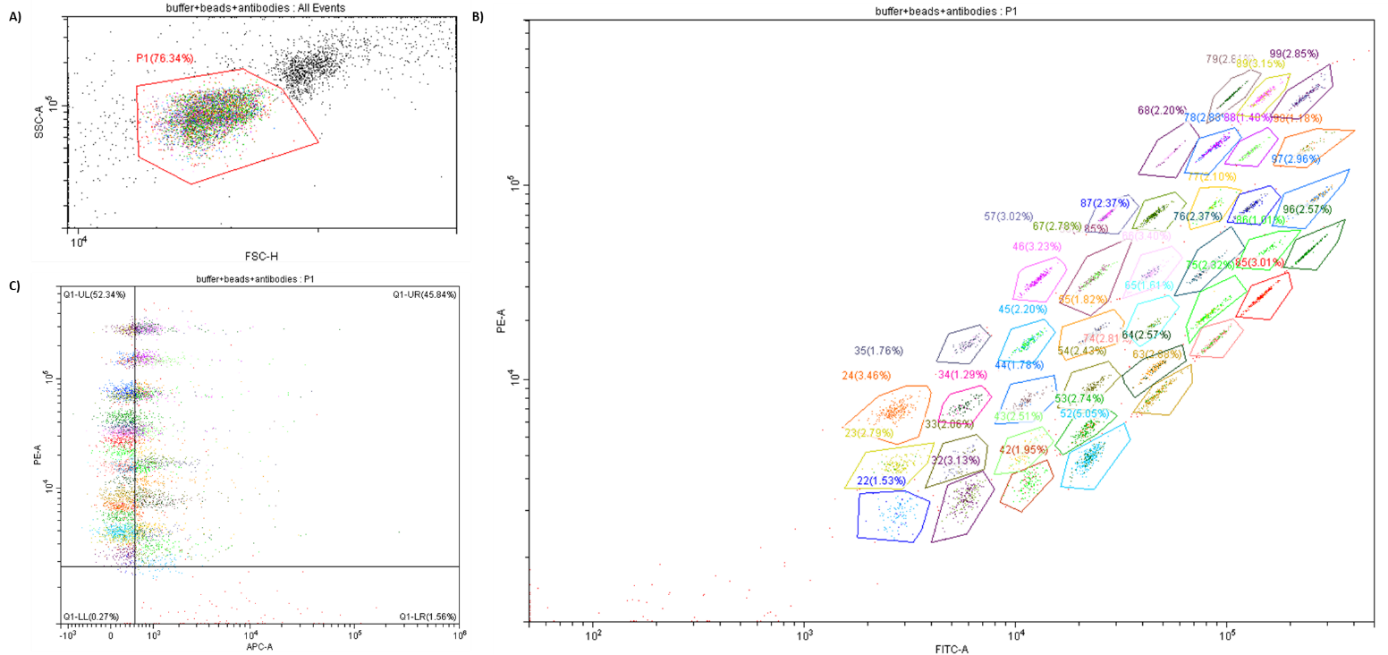
 Supplementary Figure 2.** Gating for the MACSPlex Bead based detection of exosome from Human platelet free blood plasma. (A) Singlet beads shown in detection window denoted as “P1”. (B) 39 individual gates set for the detection of 39 detection markers (all different sized beads). (C) APC-A detection threshold set for fluorescent detection.

| **Mean ± SD** | **TC** | **WT Control** | | **PCSK9-/-** | | **LDLR-/-** |
| --- | --- | --- | --- | --- | --- | --- |
|  |  |  |  | |  | |
| Mean | **Baseline** | 1.936 | 0.759 | | 5.204 | |
| SD |  | 0.045 | 0.113 | | 0.738 | |
|  |  |  |  | |  | |
| Mean | **After HFD** | 3.816 | 2.685 | | 14.454 | |
| SD |  | 0.347 | 0.513 | | 4.454 | |
|  |  |  |  | |  | |
| Mean | **Old age** | 2.192 | 0.360 | | 5.752 | |
| SD |  | 0.899 | 0.126 | | 0.328 | |
|  |  |  |  | |  | |
| **Mean ± SD** | **LDL** | **WT Control** | **PCSK9-/-** | | **LDLR-/-** | |
|  |  |  |  | |  | |
| Mean | **Baseline** | 0.490 | 0.170 | | 3.145 | |
| SD |  | 0.064 | 0.055 | | 0.980 | |
|  |  |  |  | |  | |
| Mean | **After HFD** | 1.377 | 0.528 | | 7.151 | |
| SD |  | 0.325 | 0.201 | | 2.462 | |
|  |  |  |  | |  | |
| Mean | **Old age** | 0.746 | 0.067 | | 4.896 | |
| SD |  | 0.497 | 0.025 | | 0.415 | |
|  |  |  |  | |  | |
| **Mean ± SD** | **HDL** | **WT Control** | **PCSK9-/-** | | **LDLR-/-** | |
|  |  |  |  | |  | |
| Mean | **Baseline** | 0.526 | 0.662 | | 1.310 | |
| SD |  | 0.214 | 0.114 | | 0.182 | |
|  |  |  |  | |  | |
| Mean | **After HFD** | 2.152 | 2.459 | | 2.562 | |
| SD |  | 0.610 | 0.515 | | 0.636 | |
|  |  |  |  | |  | |
| Mean | **Old age** | 0.866 | 0.187 | | 0.864 | |
| SD |  | 0.292 | 0.089 | | 0.340 | |

**Supplementary Table 3.** A table showing the raw values of cholesterol levels measured at baseline, after high-fat diet and at old age in all three mice groups. The table shows measurements of total cholesterol (TC), low-density lipoprotein cholesterol (LDL) and high-density lipoprotein cholesterol (HDL). All readings presented here are in mmol/L.


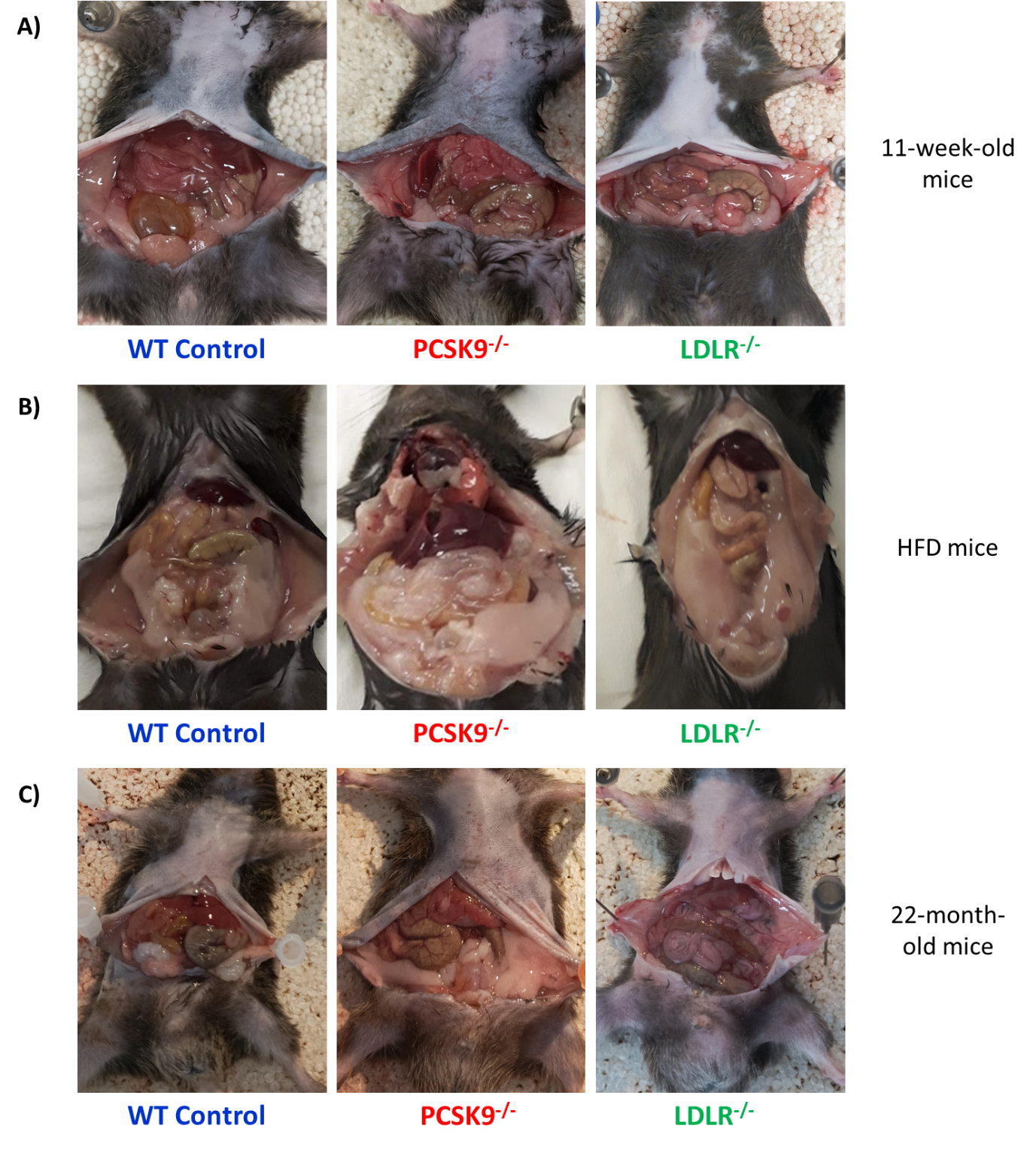
 **Supplementary Figure 3.** Open abdominal cavities of mice taken at termination of experiment displaying internal organs and visceral fatty tissue. (A) Images of opened abdominal cavities of, wild type (WT), PCSK9^−/−^, LDLR^−/−^ mice at baseline. (B) Images displaying fatty tissue in abdominal cavities of, WT, PCSK9^−/−^, LDLR^−/−^ mice after HFD. (C) Images showing opened abdominal cavities of, WT, PCSK9^−/−^, LDLR^−/−^ mice at old age.

**
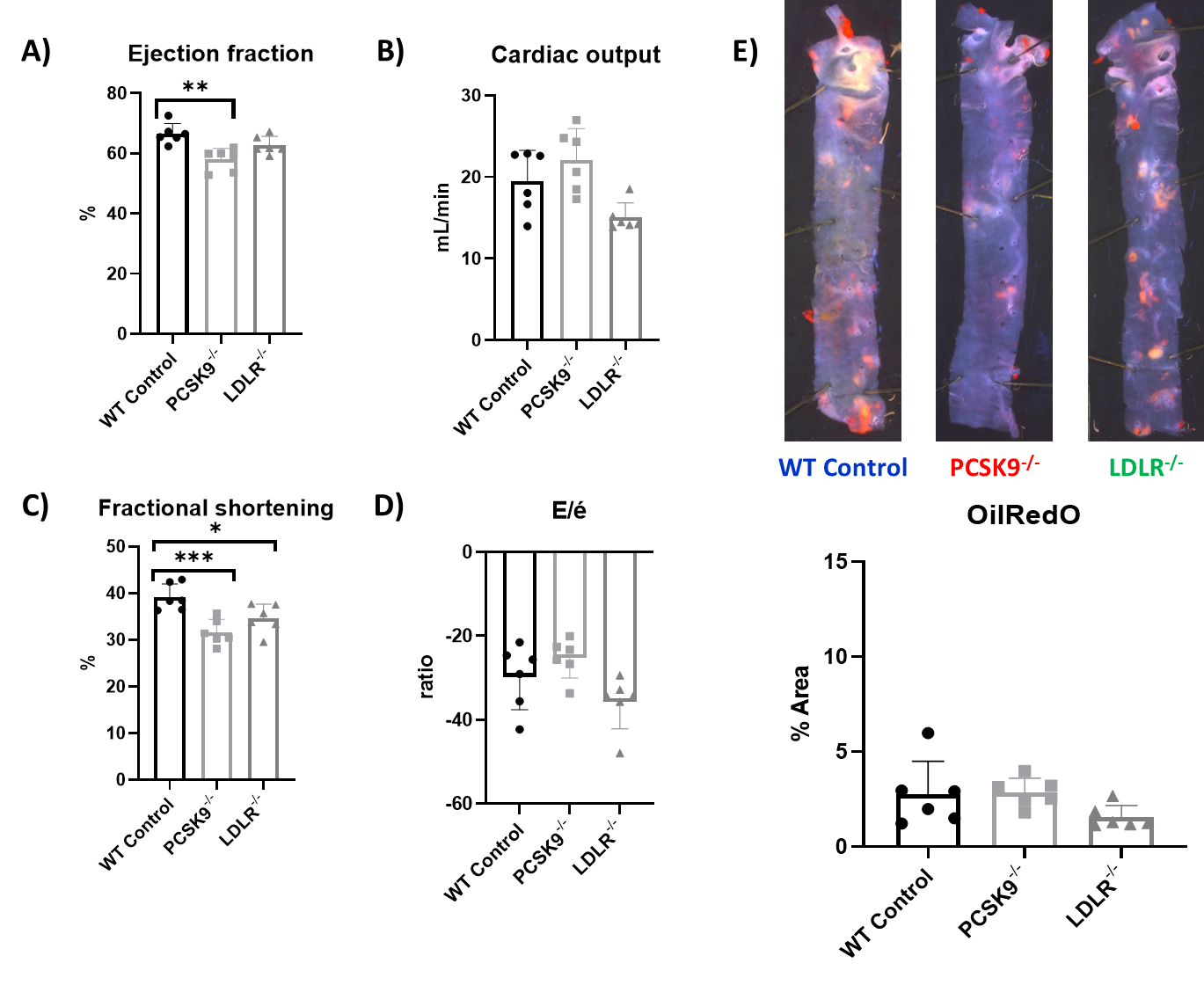
 Supplementary Figure 4.** Cardiovascular function and atherosclerotic plaques measured in the wild type (WT), PCSK9^−/−^, and LDLR^−/−^ at baseline. (A) Left ventricular ejection fraction, (B) cardiac output, (C) fractional shortening measurements, and (D) E/e’ ratio measurements. (E)Representative images of Oil Red O staining of the aortic arches and their quantifications in all three mice models, in young animals before commencement of high-fat diet.


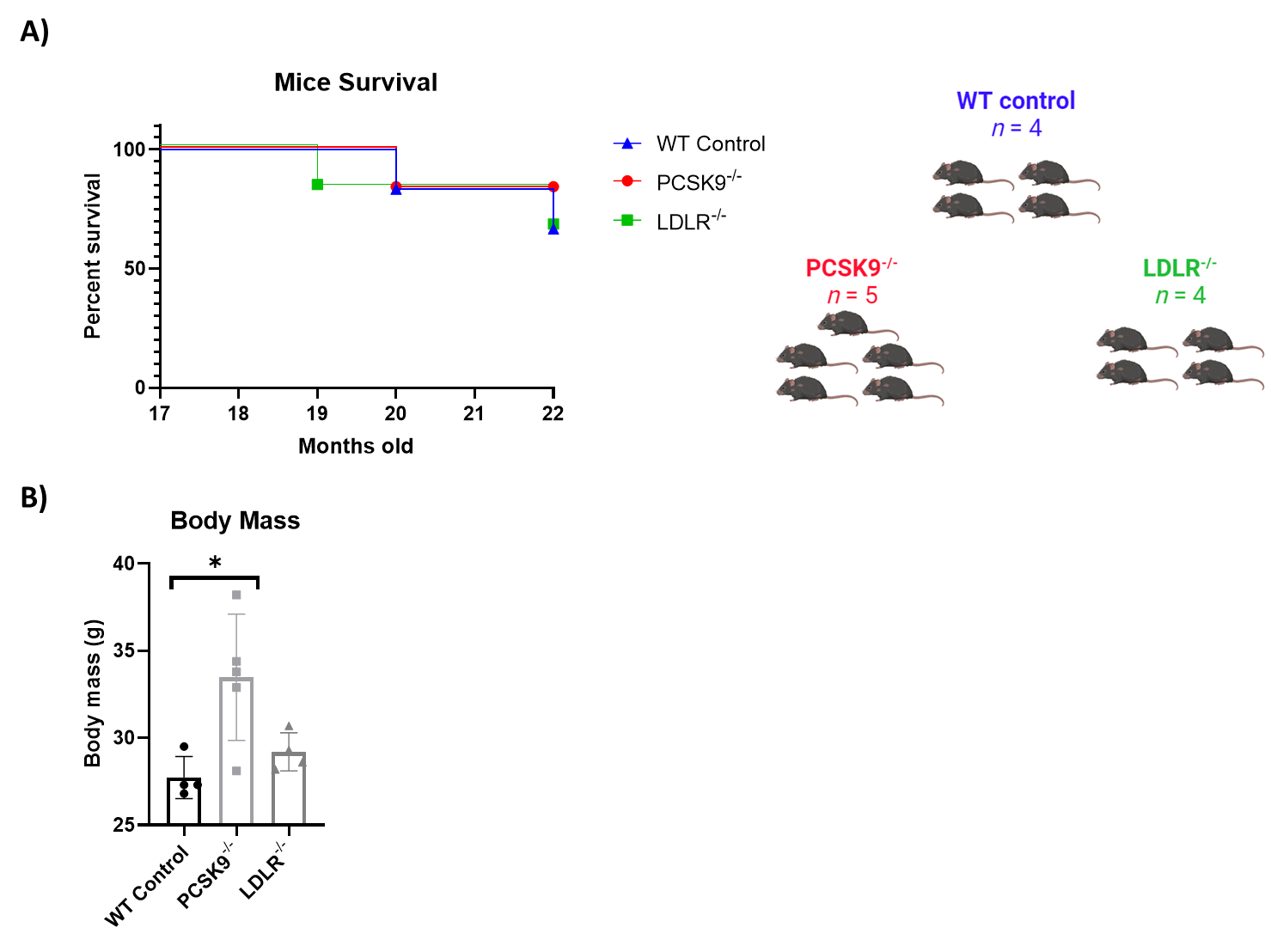
 **Supplementary Figure 5.** Additional data on old age mice showing differentiation between group survival and body mass**.** (A) A survivability curve showing the survivability of wild type, PCSK9^−/−^, and LDLR^−/−^ mice models at old age, all groups started with 6 mice. (B) Body mass of mice at old age on normal diet.


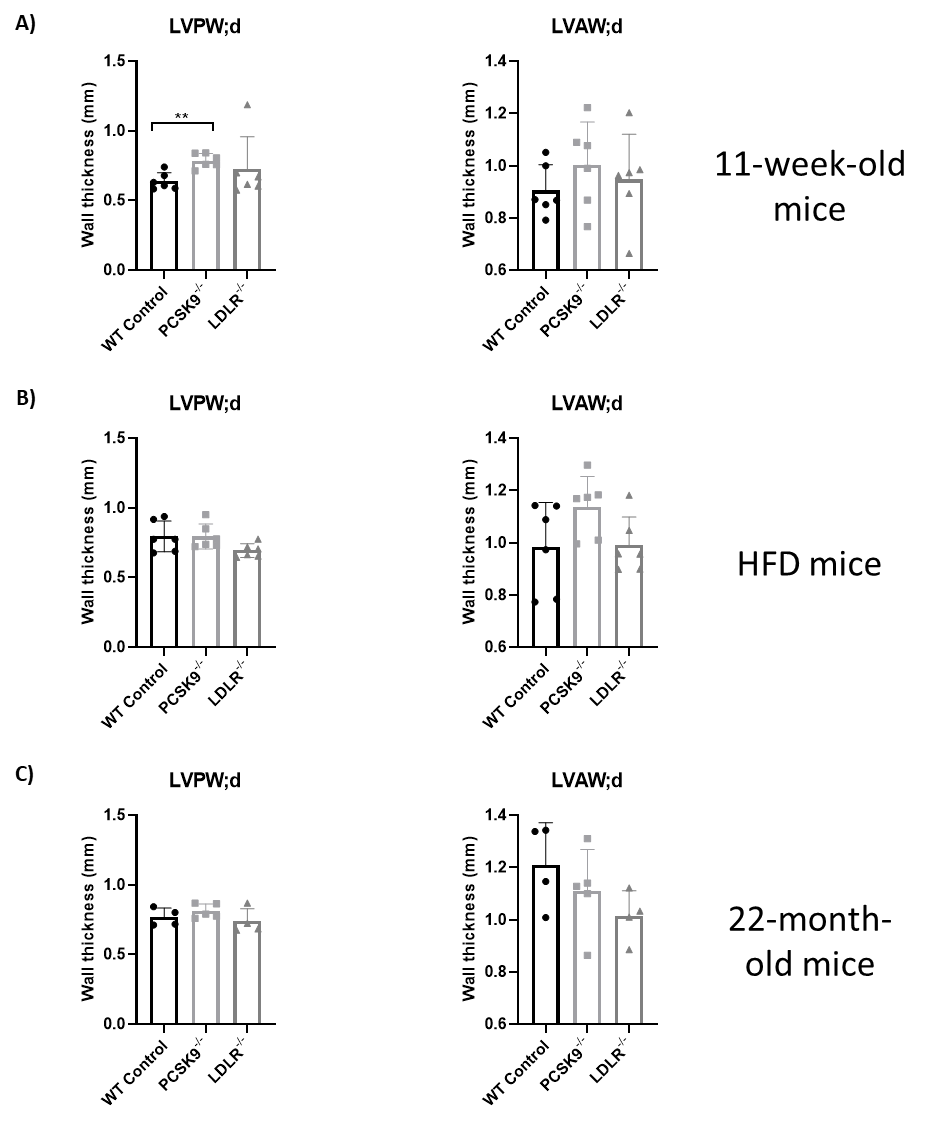


**Supplementary Figure 6.** Posterior and anterior left ventricular wall thicknesses (Denoted LVPW;d and LVAW;d respectively) measured in wild type (WT), PCSK9^−/−^, and LDLR^−/−^ mice. (A) LVPW;d and LVAW;d of PCSK9^−/−^, and LDLR^−/−^ compared to WT at baseline. (B) LVPW;d and LVAW;d of PCSK9^−/−^, and LDLR^−/−^ after HFD. (C) LVPW;d and LVAW;d of PCSK9^−/−^, and LDLR^−/−^ compared to WT in old age.


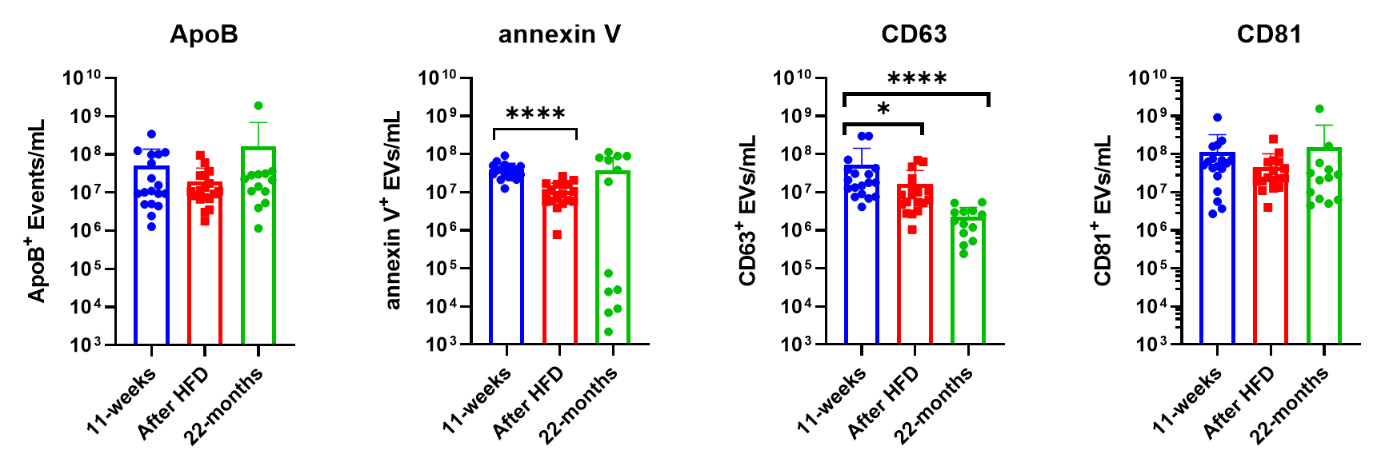


**Supplementary Figure 7**. Comparing data from all mice models together, using 11 week old mice as the baseline, here we can observe mEV changes undergone by our mouse models before versus after HFD as well as compare mEV levels of young animals (11 weeks of age) versus old mice (22 months of age).

 **Supplementary Figure 8**. The remaining 24 markers from the mouse and human MACSPlex bead-based EV array which did not show high prominence. The Allophycocyanin (APC) mean fluorescence intensity (MFI) of markers were assessed and normalised to the average of the conventional extracellular vesicle (CD9, CD63, and CD81) markers (A) Graph showing the less prominent marker levels detected in wild type, PCSK9^−/−^, and LDLR^−/−^ mice after high fat diet. (B) Graph showing the less prominent marker levels detected in patients with normal versus hypercholesterolemia.


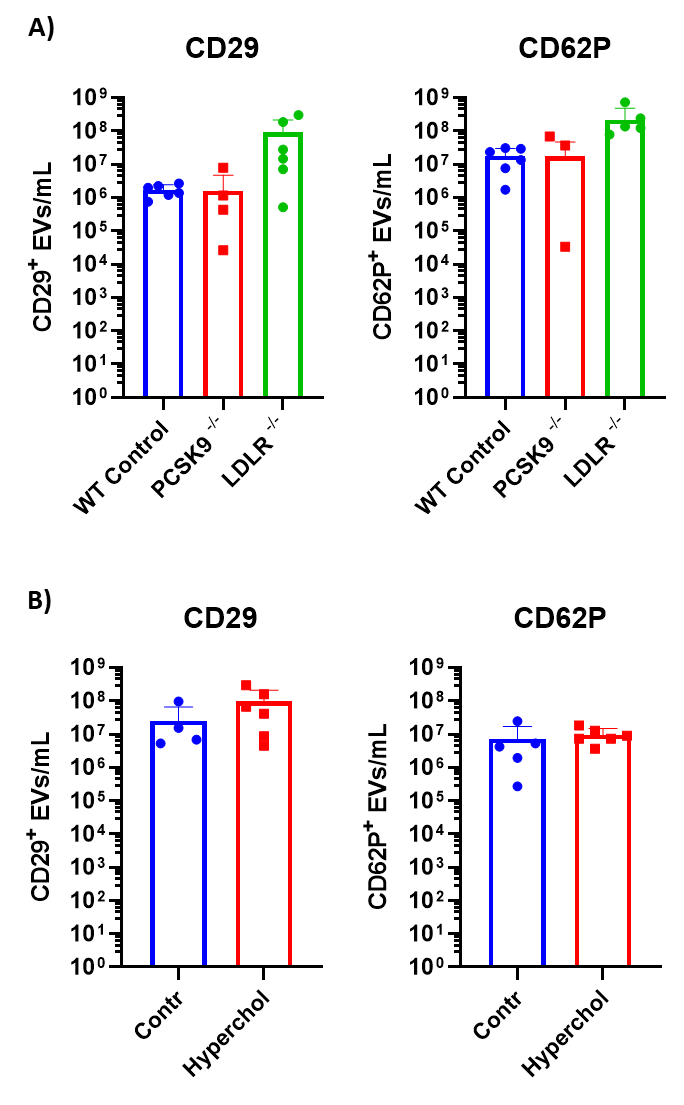


**Supplementary Figure 9.** Based on measurements with the MACSPlex kits, markers of interest were selected to test if they had significance in relation to cholesterol levels. CD29 and CD62P makers were selected to perform single EV staining using flow cytometry. (A) CD29+ and CD62P+ lEV levels in mice plasma from 3 mice groups (wild type, PCSK9-/-, and LDLR-/-) after HFD are presented. Here, no significance was detected. (B) CD29 and CD62P levels in human samples, with hypercholesterolaemia against control patients, no significance was detected here.


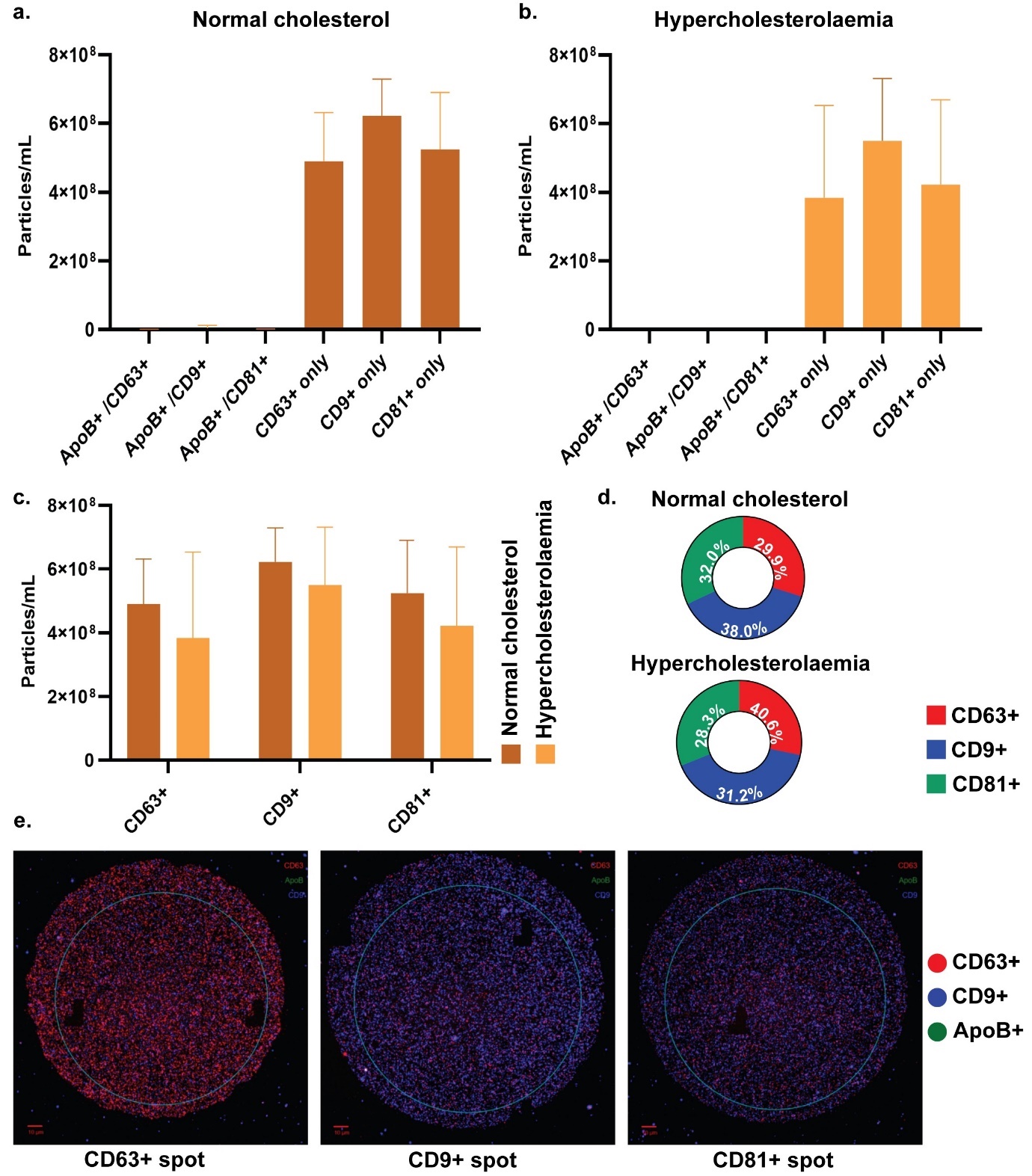


**Supplementary Figure 10**. Data gathered using SP-IRIS technology, capturing particles from human platelet free plasma (PFP) samples with exosome capture kit from Unchained Labs. Captured particles were labelled with a cocktail of CD9 (blue), CD63 (red) and ApoB (green). (A) A graph showing the positive events measured on CD63, CD9 and CD81 spots respectively, on the detection chip from patients with normal cholesterol levels. The graph also displays the detection of co-localised ApoB with CD63/CD9/CD81. (B) A graph showing the positive events measured on CD63, CD9 and CD81 spots respectively, on the detection chip from patients with hypercholesterolaemia. The graph also displays the detection of co-localised ApoB with CD63/CD9/CD81. (C) A graph displaying normal versus hypercholesteraemia patient levels of CD63, CD9 and CD81 as measured with SP-IRIS. (D) Two pie charts displaying the percentages of CD63, CD9 and CD81 captured for both of normocholesterolaemia and hypercholesterolaemia patients. (E) visual images of the major exosome detection spots (CD63, CD9, and CD81). All samples were stained with the cocktail mix of CD9 (blue), CD63 (red) and ApoB (green).

 **Supplementary Figure 11.** Gene expression levels in HUVECs after incubation with either lEVs from normocholesterolaemic or hypercholesterolaemic patients. As a positive control for key inflammatory markers lipopolysaccharide (LPS) was used, showing inflammatory response in HUVECs noted by increased CXCL1 and IL6 gene expression. All data points were normalised to a negative control (containing only HUVECs). The green dotted line at y = 2 denoting upregulation, whereas the red dotted line at y = 0.5 denotes gene downregulation.


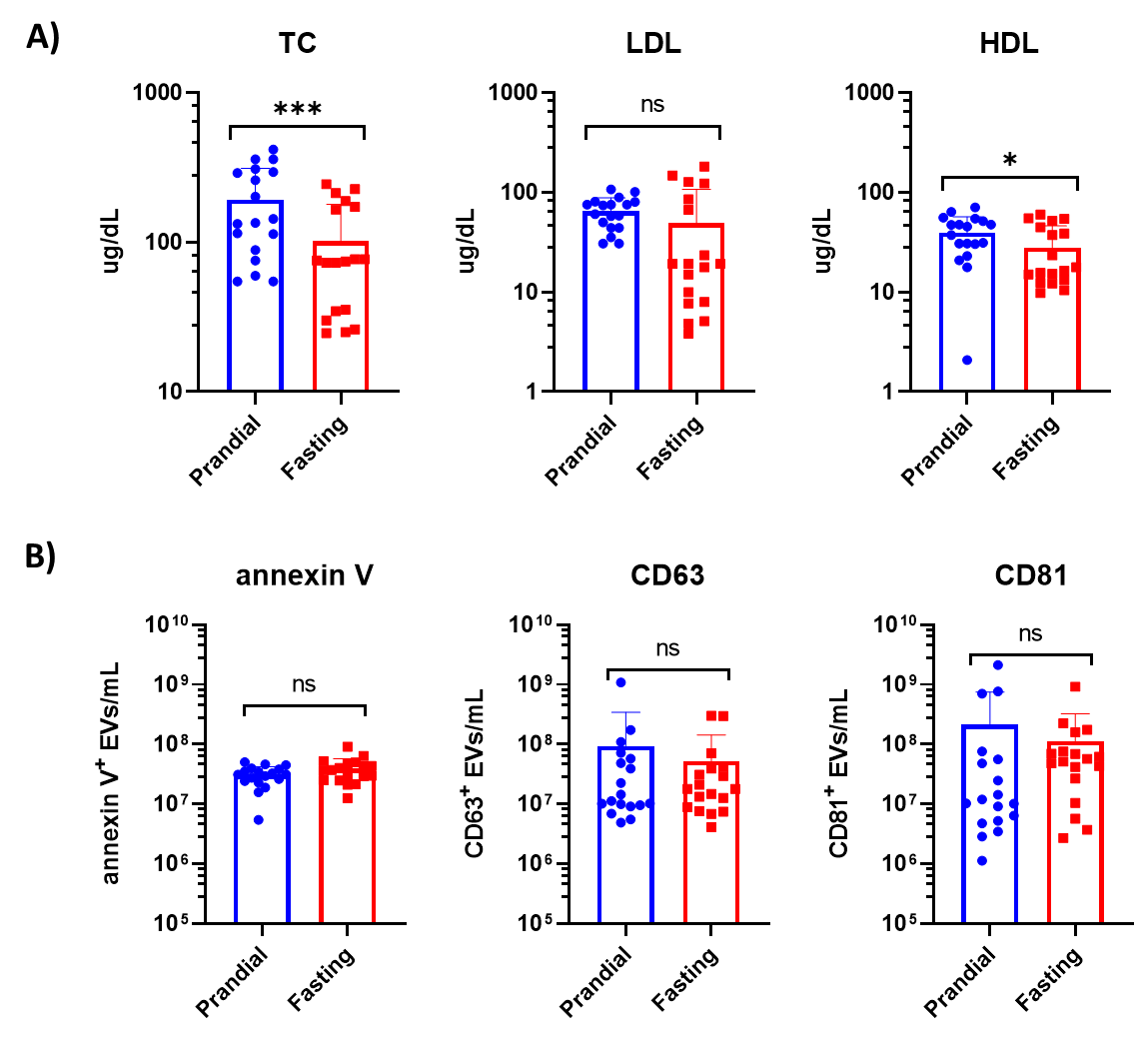
**Supplementary Figure 12**. Comparing data from all mice models together, comparing the animal groups at prandial to measurement taken after fasting. (A) Shows total cholesterol, low-density lipoprotein, and high-density lipoprotein levels after fasting, compared to prandial. (B) Three graphs show the three extracellular vesicle markers (annexin V, CD63 and CD81) of mice at prandial compared to fasting.
